# Chemical intervention of influenza virus mRNA nuclear export

**DOI:** 10.1371/journal.ppat.1008407

**Published:** 2020-04-02

**Authors:** Matthew Esparza, Amir Mor, Hanspeter Niederstrasser, Kris White, Alexander White, Ke Zhang, Shengyan Gao, Juan Wang, Jue Liang, Sei Sho, Ramanavelan Sakthivel, Adwait A. Sathe, Chao Xing, Raquel Muñoz-Moreno, Jerry W. Shay, Adolfo García-Sastre, Joseph Ready, Bruce Posner, Beatriz M. A. Fontoura

**Affiliations:** 1 Department of Cell Biology, University of Texas Southwestern Medical Center, Dallas, Texas, United States of America; 2 Simmons Comprehensive Cancer Center, University of Texas Southwestern Medical Center, Dallas, Texas, United States of America; 3 Department of Biochemistry, University of Texas Southwestern Medical Center, Dallas, Texas, United States of America; 4 Department of Microbiology, Icahn School of Medicine at Mount Sinai, New York, New York, United States of America; 5 Global Health and Emerging Pathogens Institute, Icahn School of Medicine at Mount Sinai, New York, New York, United States of America; 6 Eugene McDermott Center for Human Growth and Development, University of Texas Southwestern Medical Center, Dallas, Texas, United States of America; 7 Department of Bioinformatics, University of Texas Southwestern Medical Center, Dallas, Texas, United States of America; 8 Department of Population and Data Sciences, University of Texas Southwestern Medical Center, Dallas, Texas, United States of America; 9 Department of Medicine, Division of Infectious Diseases, Icahn School of Medicine at Mount Sinai, New York, New York, United States of America; 10 The Tisch Cancer Institute, Icahn School of Medicine at Mount Sinai, New York, New York, United States of America; Emory University School of Medicine, UNITED STATES

## Abstract

Influenza A viruses are human pathogens with limited therapeutic options. Therefore, it is crucial to devise strategies for the identification of new classes of antiviral medications. The influenza A virus genome is constituted of 8 RNA segments. Two of these viral RNAs are transcribed into mRNAs that are alternatively spliced. The M1 mRNA encodes the M1 protein but is also alternatively spliced to yield the M2 mRNA during infection. M1 to M2 mRNA splicing occurs at nuclear speckles, and M1 and M2 mRNAs are exported to the cytoplasm for translation. M1 and M2 proteins are critical for viral trafficking, assembly, and budding. Here we show that gene knockout of the cellular protein NS1-BP, a constituent of the M mRNA speckle-export pathway and a binding partner of the virulence factor NS1 protein, inhibits M mRNA nuclear export without altering bulk cellular mRNA export, providing an avenue to preferentially target influenza virus. We performed a high-content, image-based chemical screen using single-molecule RNA-FISH to label viral M mRNAs followed by multistep quantitative approaches to assess cellular mRNA and cell toxicity. We identified inhibitors of viral mRNA biogenesis and nuclear export that exhibited no significant activity towards bulk cellular mRNA at non-cytotoxic concentrations. Among the hits is a small molecule that preferentially inhibits nuclear export of a subset of viral and cellular mRNAs without altering bulk cellular mRNA export. These findings underscore specific nuclear export requirements for viral mRNAs and phenocopy down-regulation of the mRNA export factor UAP56. This RNA export inhibitor impaired replication of diverse influenza A virus strains at non-toxic concentrations. Thus, this screening strategy yielded compounds that alone or in combination may serve as leads to new ways of treating influenza virus infection and are novel tools for studying viral RNA trafficking in the nucleus.

## Author summary

Influenza virus remains a major public health threat killing ~250,000–500,000 people yearly. In pandemic years, influenza infection can lead to even higher mortality rates, as in 1918 when at least 20 million deaths occurred worldwide. Available treatments include vaccines and a few antiviral drugs, but both are limited by mutability of the virus and the development of resistance. Here we leveraged on knowledge of our previous work that uncovered a unique influenza virus pathway inside the nucleus of the host cell, which have the potential to be targeted by a novel therapeutic strategy. We designed and performed a new screen to identify chemical compounds that inhibited viral mRNA production and transport from the nucleus to the cytoplasm of the host cell. Among these compounds, we highlight a novel nuclear export inhibitor of a subset of viral and cellular mRNAs that does not substantially alter bulk cellular RNA export or levels at non-toxic dosages. Consequently, this small molecule inhibits virus replication and therefore can be used as a lead for drug development and serve as an experimental tool for further understanding key steps of the influenza virus mRNA nuclear export pathway.

## Introduction

As a major human pathogen, influenza A viruses associated mortality ranges from 291,000 to 646,000 deaths/year worldwide [1]. Available prevention measures and treatments include vaccines and a few antiviral drugs; however, both are limited by the mutability of the virus and the development of resistance [2–4]. In addition, antiviral drugs are usually most effective within the first 48 h of infection and vaccines are less effective in the elderly population. With the lack of robust and diverse medical interventions available, multiple antiviral strategies are needed to provide additional therapeutic options for influenza infections. One strategy is to identify viral-host interactions that can be targeted without compromising major host cellular functions. As the virus enters the host cell via endocytosis, the viral M2 ion channel on the viral membrane acidifies the interior of the virus particle. This enables viral uncoating and subsequent release of the viral genome into the host cytoplasm upon fusion of the viral and endosomal membranes. As the eight unique vRNPs enter the host cell nucleus, transcription initiates and 2 of the 8 viral mRNAs undergo alternative splicing. It is the alternative splicing event of the viral M1 mRNA into the viral M2 mRNA that generates the viral M2 protein that is key for viral entry [5]. The M2 protein is also important for viral budding [6, 7] and inhibition of autophagy [8]. M1 mRNA also encodes the M1 protein, which has key functions in viral intracellular trafficking and as an structural component of the infectious virions [5].

We have shown that the M1 to M2 mRNA splicing event occurs at nuclear speckles [9], which are known as storage sites for splicing and other RNA processing factors [10], and that this process requires key viral-host interactions for both splicing and nuclear export of the viral M2 mRNA [9, 11–13]. These data suggest a pathway in which the viral NS1 protein interacts with the cellular NS1-BP protein, which in turn binds hnRNP K to target the M1 mRNA from the nucleoplasm to nuclear speckles. At this nuclear body, the U1 snRNP and/or dissociation of NS1 induces a remodeling of the protein-RNA complex in a manner that hnRNP K recruits U1 snRNP to the M2 5′ splice site on M1 mRNA to mediate splicing. Then, NS1 and NS1-BP together with key members of the mRNA nuclear export machinery (the RNA helicase UAP56 and the mRNA export factor Aly/REF) promote nuclear export of M1 and M2 mRNAs [9]. Others have also reported on UAP56 function in influenza virus mRNA export [14, 15], which may be related to the recently reported interaction between UAP56 and the viral NS1 protein [16]. Additionally, UAP56 has been shown to bind the cellular antiviral MxA protein to potentially regulate antiviral response [17]. UAP56 also binds the viral NP protein to mediate viral replication coupled with encapsidation [18]. Since most splicing occurs in the nucleoplasm, this pathway through nuclear speckles might constitute a non-canonical process for splicing and nuclear export of a subset of cellular mRNAs. In fact, it has been reported that post-transcriptional splicing likely occurs at nuclear speckles [19]. A subset of poly(A) RNA has been detected at nuclear speckles and these sites were shown to mediate their nuclear export to the cytoplasm [20–24]. This is consistent with our recent data on NS1-BP interactions with key constituents of the splicing and mRNA export machineries and its function in splicing and nuclear export of a subset of viral and host mRNAs [11, 13]. For subsequent translocation through the nuclear pore complex, M mRNAs then usurp the NXF1(TAP)-mediated export pathway [25–28].

Since this splicing-export pathway through nuclear speckles does not impact bulk mRNA but only a subset of viral and cellular mRNAs [9, 13], chemical compounds that would antagonize this process have the potential of not being overly toxic and could inhibit virus replication. Thus, we performed an image-based chemical screen using single-molecule RNA-FISH to detect the viral M mRNA (M1 and M2 mRNAs) and thereby identify chemical compounds that would inhibit different steps of this speckle-export intranuclear pathway but that would not significantly compromise bulk poly(A) RNA. Indeed, we identified chemical compounds that inhibited viral mRNA processing and nuclear export, and which did not significantly affect bulk cellular mRNA levels or intracellular distribution. This selective approach offers a window of opportunity for targeting virus-host interactions, which favor the mining of compounds that target the virus with less impact to the host. Furthermore, differential requirements for nuclear export of viral mRNAs and for a subset of cellular mRNAs were revealed by one of the top hits, uncovering a new tool for studying these poorly known cell biological processes.

## Results

### High-throughput screen to identify inhibitors of viral M mRNA processing and nuclear export

**Fig 1:**
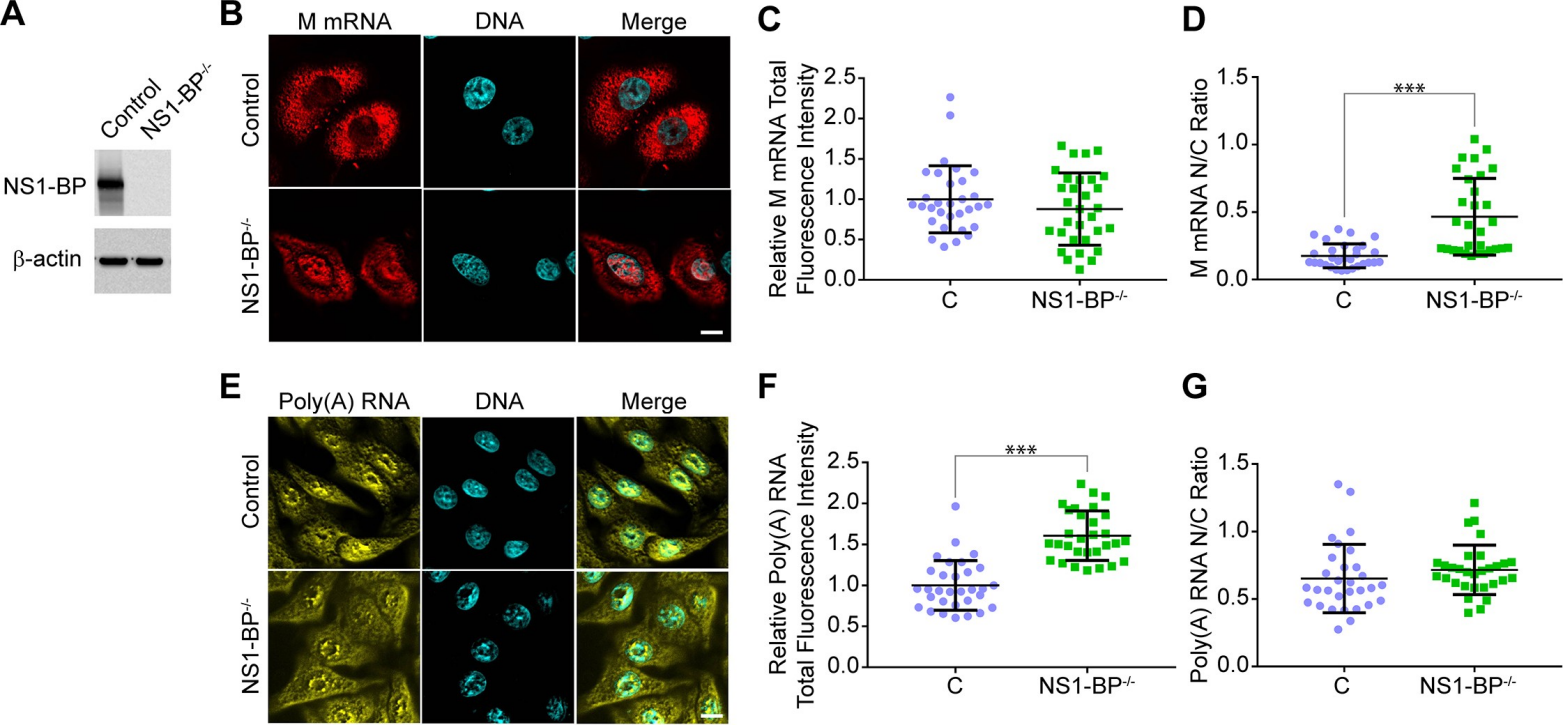
Loss of NS1-BP protein inhibits viral M mRNA nuclear export without significantly altering intracellular distribution of bulk cellular poly(A) RNA. (**A**) Disruption of the NS1-BP gene by CRISPR-Cas9 system yielded A549 cells lacking NS1-BP protein. Cell lysates from control or NS1-BP knockout cells were subjected to western blot analysis with antibodies against NS1-BP antibody or β-tubulin, as control. (**B**) Wild-type or NS1-BP^-/-^ A549 cells were infected with influenza virus (A/WSN/33) at MOI 2 for 6h. Single-molecule RNA fluorescence *in situ* hybridization (smFISH) was performed to detect influenza virus M mRNA. Hoechst staining labeled nuclei. Scale bar = 10 μm. (**C,D**) Quantification of total fluorescence intensity of M mRNA in the nucleus and cytoplasm of wild-type or NS1-BP^-/-^ cells (**C**), or nuclear-to-cytoplasmic (N/C) ratios of M mRNA in these cells (**D**) from panel B. (**E**) Non-infected wild-type or NS1-BP^-/-^ A549 cells were subjected to RNA-FISH to label poly(A) RNA. (**F,G**) Quantification of total fluorescence intensity of poly(A) RNA in the nucleus and cytoplasm of wild-type or NS1-BP^-/-^ cells (**F**), or nuclear-to-cytoplasmic (N/C) ratios of poly(A) RNA in these cells (**G**) from panel E. Thirty cells were quantified for each condition. These data are representative of three independent experiments. ***p<0.001.

We have previously reported that knockdown of the cellular NS1-BP protein inhibits influenza virus M mRNA splicing and nuclear export through host nuclear speckles [9]. We have now knocked out NS1-BP using the CRISPR/Cas9 system (Fig 1A) and these cells show a slight reduction in growth rate (S1 Fig). We then subjected wild-type and NS1-BP knockout cells to single-molecule RNA fluorescence *in situ* hybridization (smRNA-FISH) to detect influenza virus M1 mRNA in infected cells (Fig 1B–1D) and oligo-dT *in situ* hybridization to label bulk cellular poly(A) RNA in the absence of infection (Fig 1E–1G). While viral M1 mRNA nuclear export is substantially inhibited in the absence of NS1–BP (Fig 1B–1D), as expected based on our previous results with siRNA [9], bulk cellular poly(A) RNA distribution between the nucleus and cytoplasm was not altered in the absence of NS1-BP, but total intracellular levels were increased (Fig 1E–1G). These results indicate that the viral M mRNA uses a distinct mechanism to be exported from the nucleus to the cytoplasm, which is not shared by the bulk of the cellular mRNA. Thus, we postulated that it should be possible to identify specific inhibitors of this unique mechanism that would impact nuclear export of the influenza virus M RNA without significantly affecting bulk cellular RNA processing and expression.

**Fig 2:**
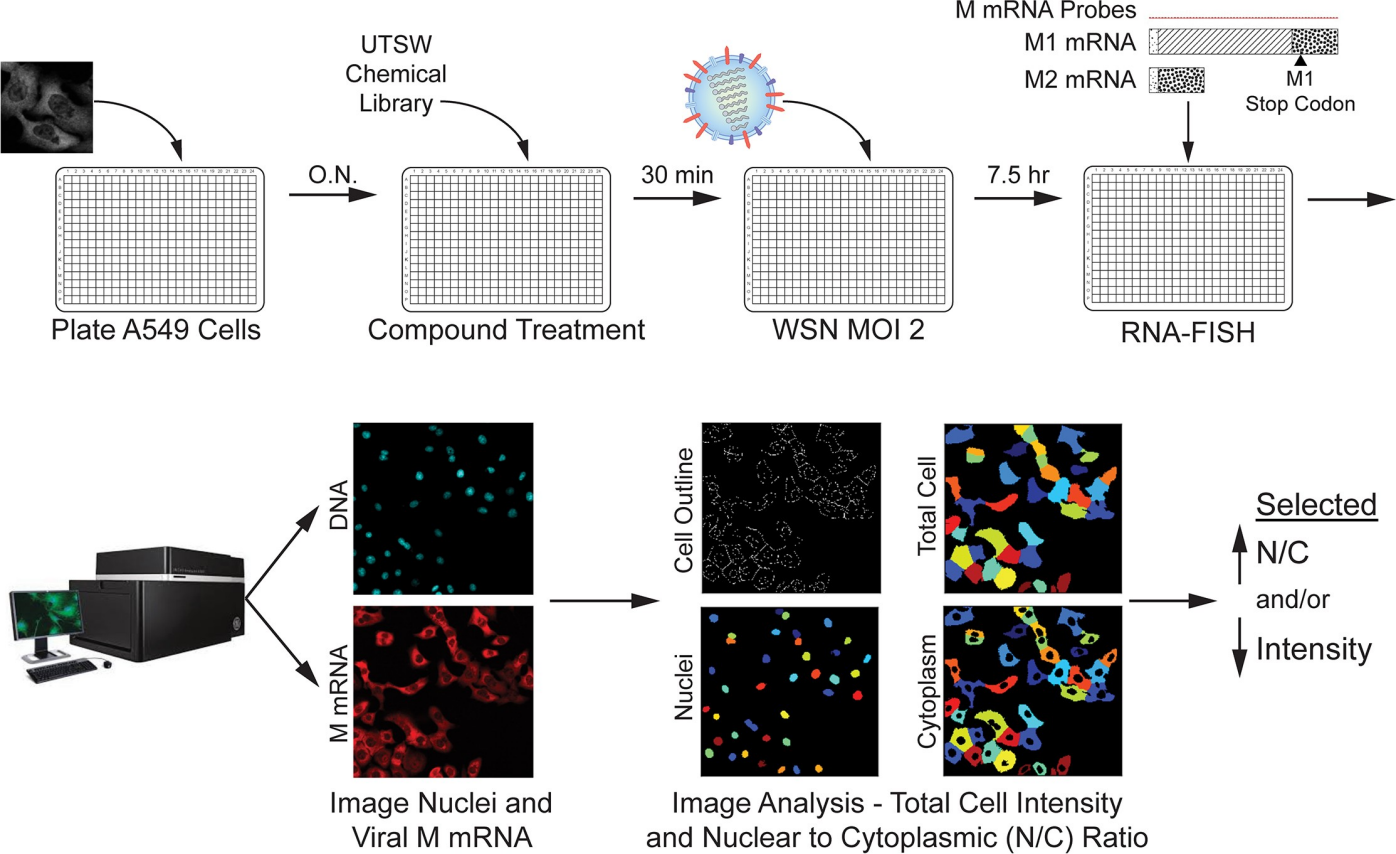
Schematic representation of a high-throughput screen to identify chemical inhibitors of viral M mRNA processing and nuclear export. Screen was performed using a chemical library of 232,500 compounds in A549 cells. Cells were incubated with compounds for 30 min and, for robust imaging analysis, ~ 100% of the cells were infected with influenza virus (WSN), at MOI 2 for 7.5h. Viral M mRNA was detected by smRNA-FISH and images were systematically taken in a high throughput microscope (IN Cell Analyzer 6000). Samples on 384-well black clear-bottom plates were imaged at 20X magnification using the Hoechst and dsRed filters. 4 fields of view per well were collected for each channel. The distribution of fluorescent signal between the nucleus (N) and the cytoplasm (N/C ratio) as well as total cell signal intensity were quantified using GE IN Cell Analyzer Workstation (version 3.7.3) and Pipeline Pilot (version 9.5; Biovia). Data was imported into the GeneData’s Screener^™^ software analysis suite for quality control to ensure that data quality is high for all plates in each experimental run (Z’ > 0.4).

**Fig 3:**
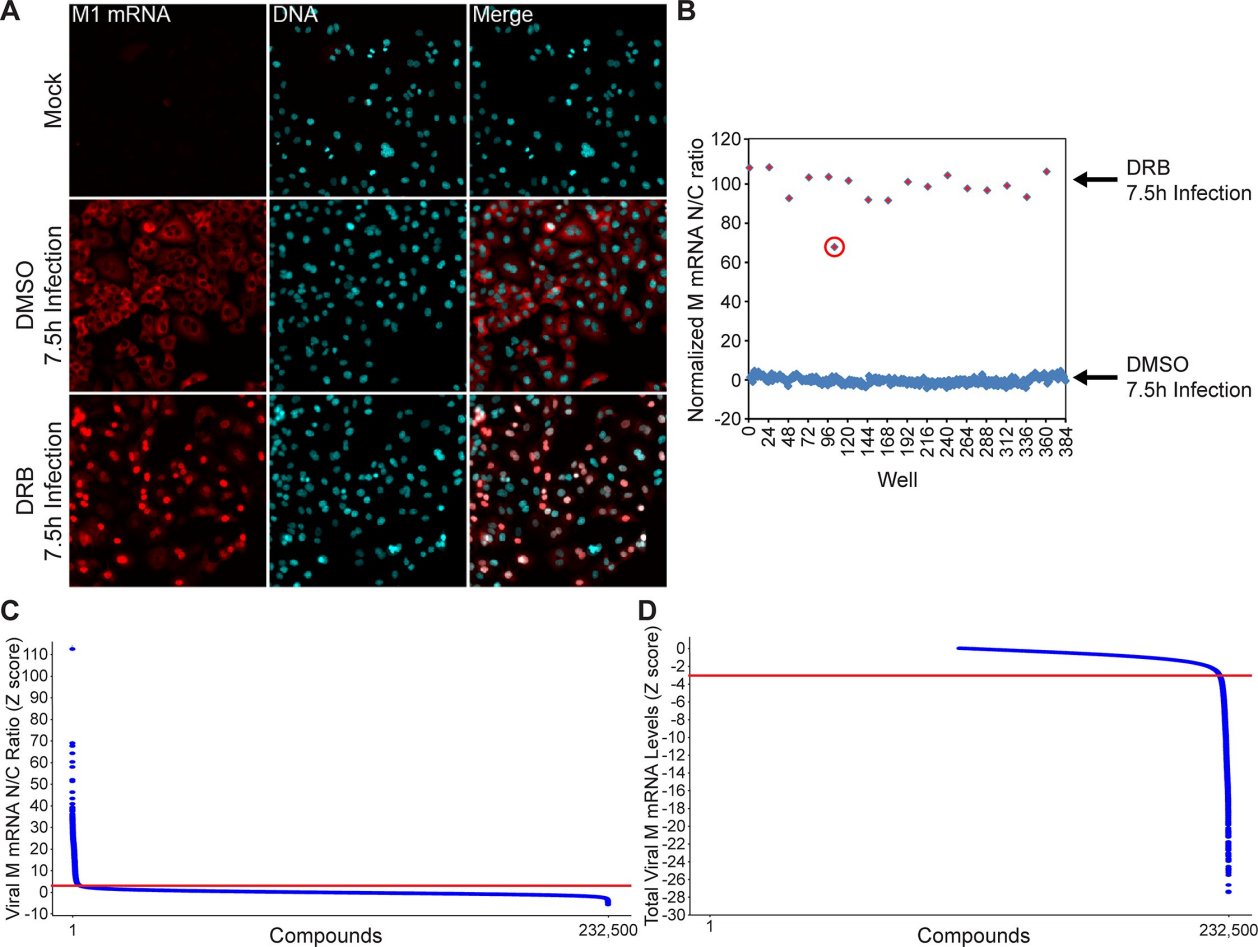
Identification of small molecules that inhibit viral M mRNA nuclear export and/or decrease viral M mRNA levels. (**A**) Representative images showing uninfected cells; cells infected with A/WSN/33 and pretreated with 0.5% DMSO (control), which show viral M mRNA exported into the cytoplasm, in red; and cells infected with A/WSN/33 pre-treated with 2.5 μM of the transcription inhibitor DRB (5,6-dichloro-1-β-D-ribofuranosylbenzimidazole). DRB served as positive control for viral M mRNA nuclear retention. (**B**) Distribution of nuclear (N) to cytosolic (C) (N/C ratio) of all wells in a mock assay plate showing the assay window and sensitivity. DMSO wells were normalized to 0 and DRB positive control wells were normalized to 100. The circled red diamond shape represents a lower dose of DRB and shows lower normalized N/C ratio than the other diamonds representing the full control dose of DRB. (**C**) Rank-sorted Z-score of the Nuclear to Cytoplasmic (N/C) ratio of viral M mRNA in A549 cells after individual treatment with 232,500 compounds (2.5 μM). Each N/C value is expressed as a Z-score, indicating the number of standard deviations from the median plate ratio. Points above the red line at Z-score 3 represent compounds that were considered hits in the primary screen. (**D**) Rank-sorted Z-score of the total intensity of viral M mRNA after compound treatment. Each value is expressed as a Z-score, indicating the number of standard deviations from the median plate intensity. Points below the red line at Z-score -3 represent compounds selected as hits in the primary screen. To better visualize the distribution of compounds within the desired range (Z-score < -3), the Z-score range of the graph has been focused to view data points that show decrease in viral M mRNA fluorescence.

**Fig 4:**
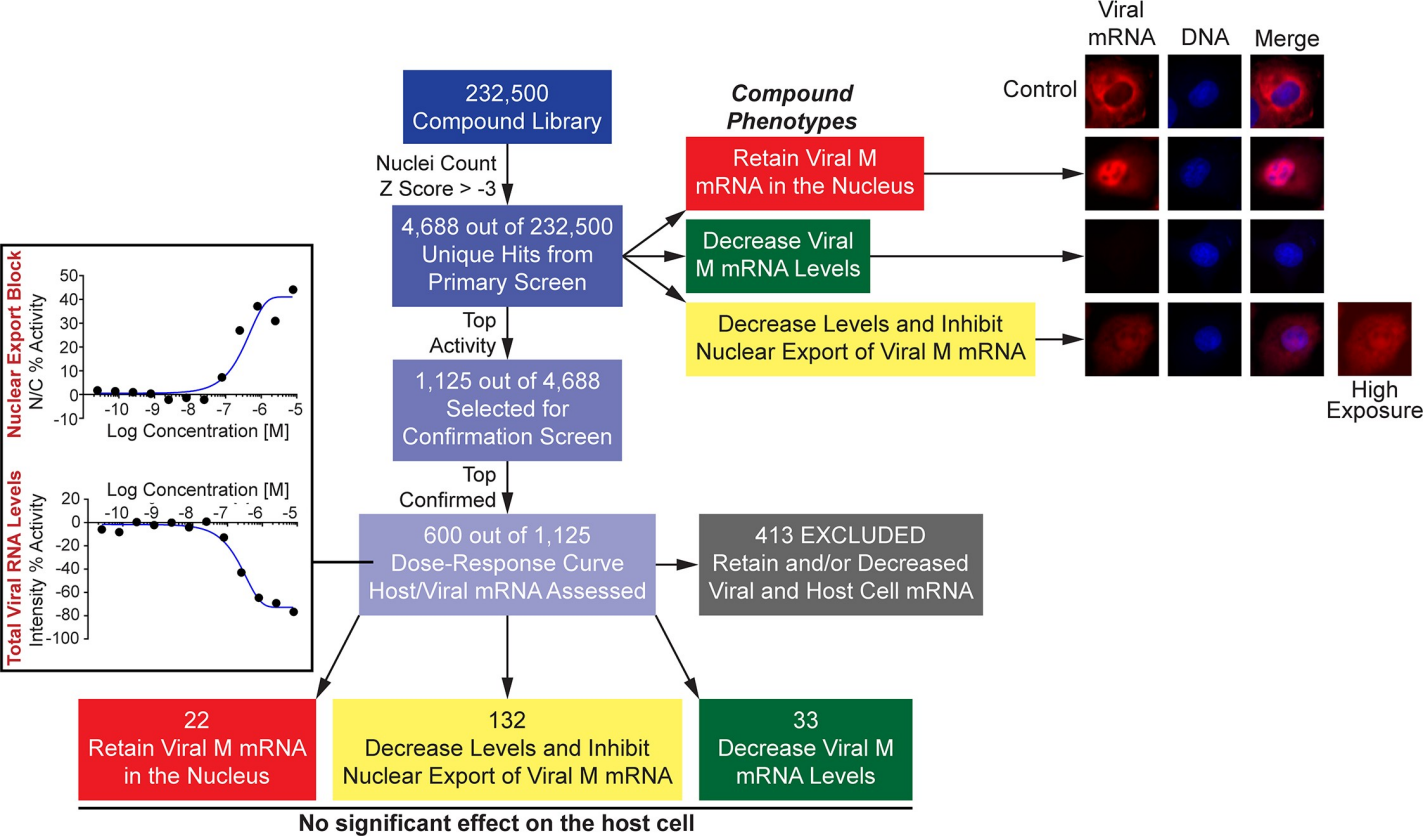
Schematic representation of identification and selection of top hits that inhibit viral M mRNA nuclear export and/or expression. Out of the 232,500 compounds tested in the primary screen, we selected compounds with Z-scores ≥ 3 for the N/C ratio and compounds that decreased viral mRNA levels with Z-scores ≤ -3. Compounds that reduced nuclear count significantly (Z-score < -3) were considered cytotoxic and were eliminated from further consideration. Of those remaining, the 1,125 compounds with the highest Z-scores were chosen for confirmation studies. The top 600 compounds from single-dose confirmation studies were further evaluated in a 12-point dose response study to assess the potency (AC50 –concentration at 50% activity). Examples of dose-response curves showing phenotypes of hits that induced viral M mRNA nuclear export block (increased N/C) and decrease in viral M mRNA levels (decreased intensity) are depicted. During this step, bulk cellular poly(A) RNA localization and intensity were also assessed by smRNA-FISH to determine the effect of these compounds on host cell mRNA (intensity and N/C ratio). Compounds that inhibited viral mRNA nuclear export and/or decreased viral mRNA levels but had no significant effect on the host cell poly(A) RNA were then selected for additional assays.

Next, we performed a high-throughput screen to select inhibitors of viral M mRNA processing and nuclear export. By adapting our previously reported protocol to visualize the M mRNAs during virus infection [9], we designed a high-throughput screening assay to identify compounds that alter M mRNA expression and trafficking without significantly compromising bulk cellular poly(A) RNA levels or intracellular distribution. The high throughput screen was performed using a chemical library of 232,500 compounds. As shown in Fig 2, cells were incubated with compounds and then infected with influenza virus (WSN) for 7.5 h. Cells were then subjected to smRNA-FISH and images were analyzed by quantifying the distribution of fluorescence signal between the nucleus (N) and the cytoplasm (N/C ratio) as well as total cell fluorescence intensity. In a control experiment, N/C ratios were identified for DMSO negative-control and DRB (5,6-dichloro-1-beta-*D*-ribofuranosylbenzimidazole) positive control (Fig 3A and 3B), which inhibits cellular processive transcription by RNA polymerase II and also prevents nuclear export of a subset of influenza virus mRNAs, including M mRNA [29]. Compounds with high N/C ratios (Z-score ≥ 3 compared to the robust test population median on each plate), indicating nuclear export block of viral M mRNA (Fig 3C), were selected for follow-up screening. In addition, our screen revealed compounds that selectively decreased viral RNA signal (fluorescence intensity), indicating down-regulation of viral M mRNA levels (Z-score ≤ -3 compared to the median of the test population on each plate, Fig 3D). Furthermore, we have also identified compounds that inhibited both viral M mRNA nuclear export and decreased total viral M mRNA levels (Fig 4). Compounds that reduced nuclei count were considered cytotoxic (Z-score threshold < -3, See Fig 4). In total, 4,688 of the 232,500 compounds tested were hits in the primary screen. The 4,688 compounds were clustered based on chemical structure and the 1,125 compounds (824 that inhibited M mRNA nuclear export and 301 that decreased M mRNA levels) with the highest Z-scores from each cluster were chosen for confirmation. The top 600 compounds, including both phenotypic classes, were then subjected to dose response assays to determine their potency. At this stage, we also assessed poly(A) RNA by RNA-FISH to detect potential compound effects on host bulk poly(A) RNA levels or nuclear export. Compounds that altered bulk poly(A) RNA were excluded (AC_50_ < 8 μM). Thus, only compounds that blocked viral M mRNA nuclear export or biogenesis and did not substantially affect host bulk mRNA, at non-toxic concentrations, were selected. 413 of the 600 compounds altered bulk poly(A) RNA and were excluded, thus leaving a total of 187 compounds for follow-up studies (Fig 4). Importantly, these 187 confirmed hits represent 187 structurally diverse clusters, and each cluster contains the top hit and related less active analogs (S2 Fig).

### Selective inhibition of viral mRNA nuclear export

**Fig 5:**
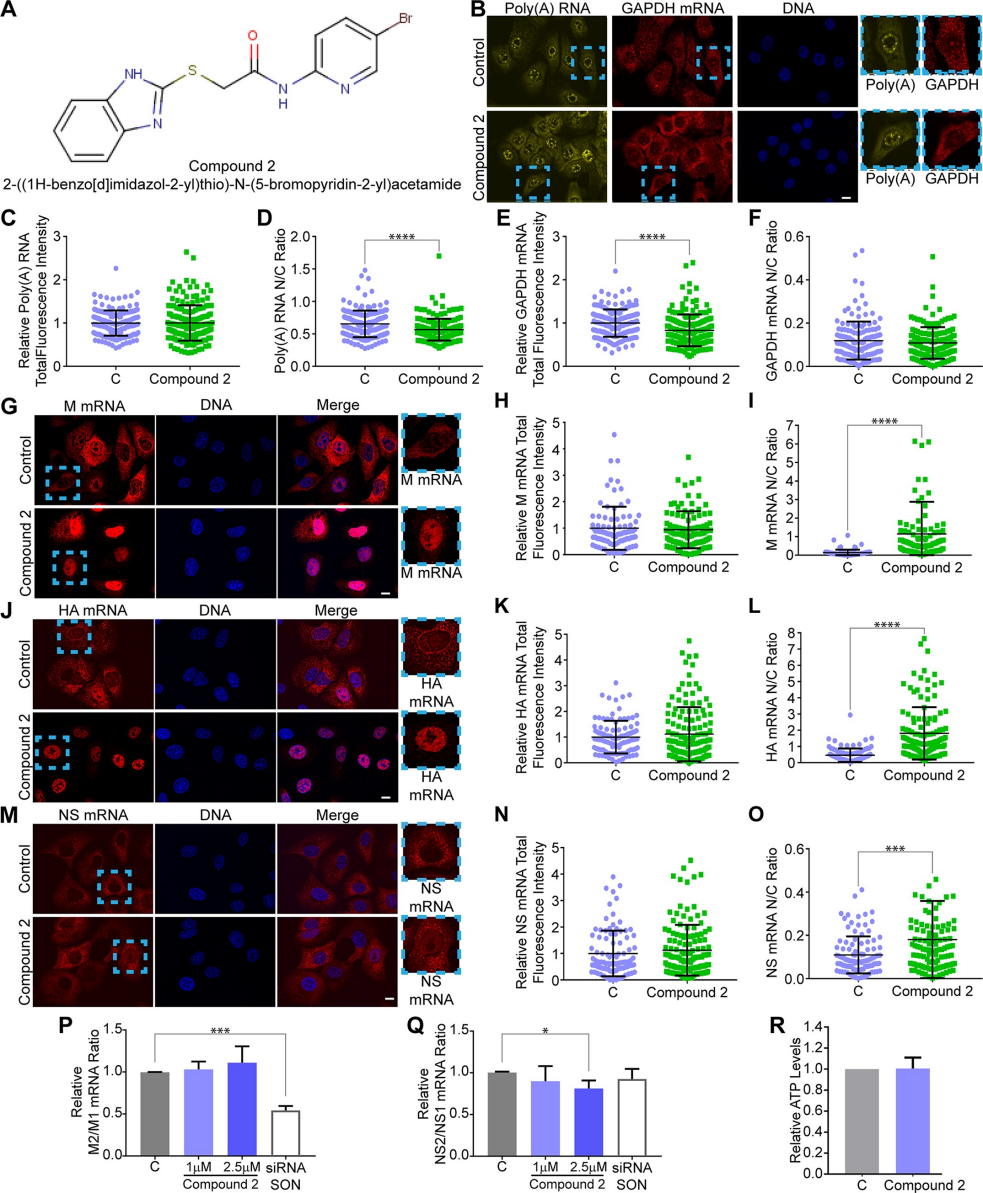
Compound 2 inhibits viral mRNA nuclear export. (**A**) Structure of compound 2. (**B**) RNA-FISH and smRNA-FISH followed by fluorescence microscopy were performed in cells treated with 0.1% DMSO or 2.5 μM compound 2 to detect poly(A) RNA and GAPDH mRNA, respectively, in uninfected cells. (**C-F**) Total fluorescence intensity or nuclear to cytoplasmic fluorescence intensity (N/C ratio) were quantified for poly(A) RNA and GAPDH mRNA in the absence or presence of compound 2. For **C** (C, *n =* 174 cells; Compound 2, *n =* 181), **D** (C, *n =* 172 cells; Compound 2, *n =* 181 cells), **E** (C, *n =* 166 cells; Compound 2, *n =* 181 cells), and **F** (C, *n =* 151 cells; Compound 2, *n =* 160 cells). (**G**) Cells were treated as in **B** except that smRNA-FISH was performed with probes to detect M mRNA in cells infected with WSN at MOI 2 for 8 h. (**H,I**) Total fluorescence intensity or nuclear to cytoplasmic fluorescence intensity (N/C ratio) were quantified for M mRNA in the absence or presence of compound 2. For **H** (C, *n =* 91 cells; Compound 2, *n =* 104 cells) and **I** (C, *n =* 101 cells; Compound 2, *n =* 95 cells). (**J**) Cells were treated as in **G** except that smRNA-FISH was performed with probes to detect HA mRNA. (**K,L**) Total fluorescence intensity or nuclear to cytoplasmic fluorescence intensity (N/C ratio) were quantified for HA mRNA in the absence or presence of compound 2. For **K** (C, *n =* 104 cells; Compound 2, *n =* 137 cells) and **L** (C, *n =* 101 cells; Compound 2, *n =* 126 cells). (**M**) Cells were treated as in **G** except that smRNA-FISH was performed with probes to detect NS mRNA. (**N,O**) Total fluorescence intensity or nuclear to cytoplasmic fluorescence intensity (N/C ratio) were quantified for M mRNA in the absence or presence of compound 2. For **N** (C, *n =* 96 cells; Compound 2, *n =* 135 cells), and **O** (C, *n =* 106 cells; Compound 2, *n =* 113 cells). At least three independent experiments were performed for each imaging analysis. (**P,Q**) Relative mRNA ratios of M2 to M1 (**P**) and NS2 to NS1 (**Q**) were determined by qPCR from RNA obtained from cells infected as in **G** and treated with 0.1% DMSO, 1 μM, or 2.5 μM compound 2. The nuclear speckle assembly factor SON was knocked down with siRNAs as positive control for inhibition of M1 to M2 mRNA splicing. Three independent experiments were performed. C, control. (**R**) Cellular ATP levels were measured in cells treated with 0.1% DMSO or 2.5 μM of compound 2 at 24 h. Four independent experiments were performed and each contained 6 technical replicates. Graphs shows data points and mean +/- SD. *p<0.05; ***p<0.001, ****p<0.0001.

For follow-up studies, we first selected compounds with the lowest AC_50_ in dose-response curves that showed retention of viral M mRNA in the nucleus by measuring nuclear to cytoplasmic ratios as in Fig 4. Among the top hits is compound 2, which is a 2-((1H-benzo[d]imidazole-2-yl)thio)-N-(5-bromopyridin-2-yl) acetamide (Fig 5A). We re-tested this compound in smRNA-FISH to confirm the intracellular distribution of viral M mRNA and also extended our analysis to other influenza virus mRNAs, including HA and NS, as well as bulk poly(A) RNA and cellular GAPDH mRNA. Image quantification was performed by determining the mRNA fluorescence intensity in whole cells or in the nucleus and cytoplasm, which is expressed as N/C ratios. We found that compound 2 did not affect the total levels of bulk cellular poly(A) RNA (Fig 5B and 5C) and slightly decreased its nuclear to cytoplasmic ratio (Fig 5B and 5D). The total levels of cellular GAPDH mRNA were also slightly decreased by compound 2 (Fig 5B and 5E) and its nuclear to cytoplasmic distribution was not affected (Fig 5B and 5F). Thus, compound 2 slightly promoted cellular poly(A) RNA export. In contrast, compound 2 robustly inhibited nuclear export of viral M mRNA (Fig 5G–5I) and HA mRNA (Fig 5J–5L). Compound 2 did not alter total M mRNA fluorescence intensity (Fig 5G and 5H) but induced nuclear retention of M mRNA (Fig 5G and 5I). A similar result was obtained for the HA mRNA (Fig 5J–5L). The total levels of the NS mRNA were not altered by compound 2 (Fig 5M and 5N), but this compound induced a weak nuclear retention of NS mRNA (Fig 5M and 5O) as compared to the effective inhibition of M and HA mRNAs (Fig 5G–5L). These results highlight differences in requirements for nuclear export of specific influenza virus mRNAs. To assess whether compound 2 had any effect on M1 to M2 splicing, we quantified the relative ratio of M2 to M1 mRNAs in the absence or presence of compound 2 by qPCR. We found no effect of compound 2 on M1 to M2 splicing as opposed to knockdown of the splicing co-factor and nuclear speckle assembly factor SON, which was used as a positive control (SON promotes M1 to M2 splicing at nuclear speckles [9]; See Fig 5P). In addition, compound 2 only slightly inhibited NS1 to NS2 splicing (Fig 5Q). Cellular ATP levels were also assessed as a surrogate for cytotoxicity and showed no significant change in ATP levels (Fig 5R). Thus, these findings indicate that compound 2 robustly targets nuclear export of a subset of mRNAs at non-toxic concentrations.

### Compound 2 phenocopies down-regulation of the mRNA export factor UAP56

**Fig 6:**
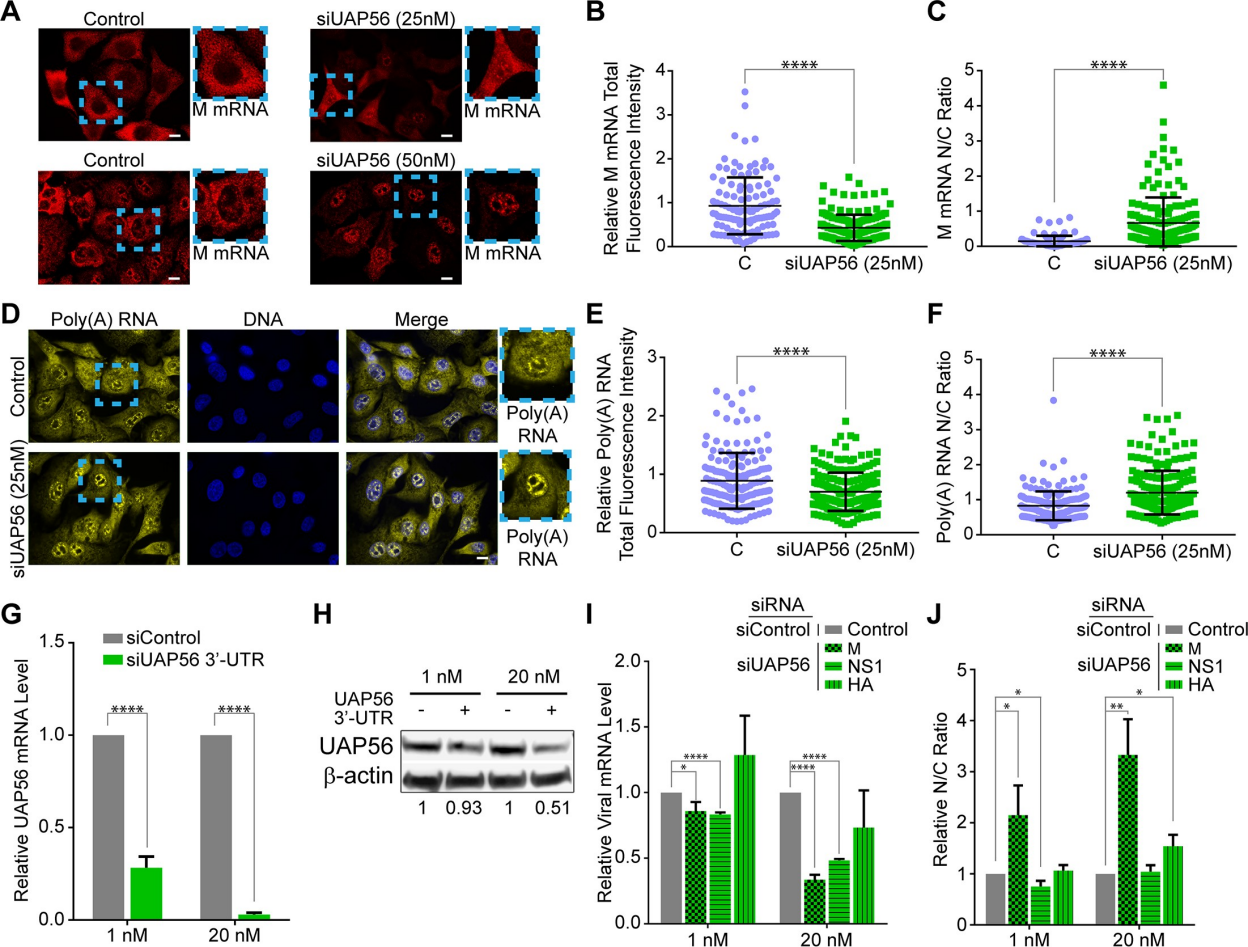
Partial depletion of the mRNA export factor UAP56 show differential export of viral mRNAs similar to compound 2. (**A**) smRNA-FISH followed by fluorescence microscopy was performed to detect M mRNA in A549 cells treated with control siRNA or with two concentrations (25 nM and 50 nM) of siRNAs that target the coding region of UAP56 or control siRNA followed by infection with WSN at MOI 2 for 8h. (**B)** Total fluorescence intensity or nuclear to cytoplasmic fluorescence intensity (N/C ratio) (**C**) were quantified for images in **A** in which cells were treated with 25 nM siRNA targeting UAP56. For **B** (C, *n =* 117 cells; siRNA UAP56, *n =* 171 cells) and **C** (Control, *n =* 97 cells; siRNA UAP56, *n =* 166 cells). Graphs show data points and mean +/- SD. ****p<0.0001. (**D**) A549 cells were treated with 25 nM siRNA targeting UAP56 or control siRNA as in **A**. RNA-FISH was performed to detect poly(A) RNA. Total fluorescence intensity (**E**) or nuclear to cytoplasmic fluorescence intensity (**F**) were quantified for images in **D**. For (**E**) (C, *n =* 171 cells; siRNA UAP56, *n =* 213 cells) and **F** (Control, *n =* 172 cells; siRNA UAP56, *n =* 208 cells). Graphs show data points and mean +/- SD. ****p<0.0001. (**G-J**) A549 cells were treated with 1 nM or 20 nM siRNA targeting the 3’UTR of the UAP56 mRNA or with control siRNA and then infected with WSN at MOI 2 for 8h. (**G**) Purified RNA from total cell lysates was subjected to qPCR to measure UAP56 mRNA levels. (**H**) Cell lysates were also subjected to western blot to detect UAP56 protein and β-actin as control. Quantification of protein bands normalized to their loading control is shown at the bottom of the blots. (**I**) Purified RNA from total cell lysates in (**G**) were subjected to qPCR to measure viral mRNA levels. (**J**) Purified RNA from nuclear and cytoplasmic fractions from cells treated as in (**G-J**) were subjected to qPCR to measure viral mRNA levels in both fractions and determine their nuclear to cytoplasmic ratios (N/C). Control for cell fractionation is shown in S3 Fig. *n* = 3. Graphs are mean +/- SD. *p<0.05, **p<0.01, ****p<0.0001.

We then tested the effect of compound 2 on the M mRNA nuclear export pathway. We and others have previously shown that M mRNA nuclear export is inhibited by knockdown of the mRNA export factor UAP56 [9, 14, 30]. This effect is also shown here with increasing concentrations of siRNAs that target UAP56 (Fig 6A–6C), emphasizing that UAP56 is critical for M mRNA nuclear export. When UAP56 mRNA was knocked down with 25 nM siRNA, a slight reduction in total levels of bulk poly(A) RNA (Fig 6D and 6E) and partial inhibition of bulk cellular poly(A) RNA nuclear export (Fig 6D and 6F) were detected. We then analyzed the total levels and intracellular distribution of viral M, HA, and NS1 mRNAs upon depletion of UAP56 with low concentrations of siRNA, which reduced UAP56 mRNA and protein levels in a dose-dependent manner (Fig 6G and 6H). Upon UAP56 depletion, purified RNA from total cell extracts, nuclear and cytoplasmic fractions were subjected to qPCR (controls for cell fractionation are shown in S3 Fig). Knockdown of UAP56 with 1 nM siRNA only slightly reduced the total levels of M and NS1 mRNAs and did not affect the levels of HA mRNA (Fig 6I). However, this level of UAP56 down-regulation was sufficient to significantly block M mRNA in the nucleus while the intracellular distribution of NS1 and HA mRNAs were not affected (Fig 6J). When the siRNA concentration targeting UAP56 was increased to 20 nM, total levels of M and NS1 mRNAs were reduced but HA mRNA level was not altered (Fig 6I). Nevertheless, M mRNA nuclear export was further blocked, HA mRNA export is also inhibited, and no effect was observed with NS1 mRNA (Fig 6J). This preferential blockage of M and HA mRNAs by partial depletion of UAP56 is similar to compound 2 effect on viral mRNA export (Fig 5G–5O). A similar pattern of preferential viral mRNA export upon UAP56 depletion has been previously described [14], but high levels of UAP56 siRNA have been shown to inhibit NS1 mRNA export [30].

**Fig 7:**
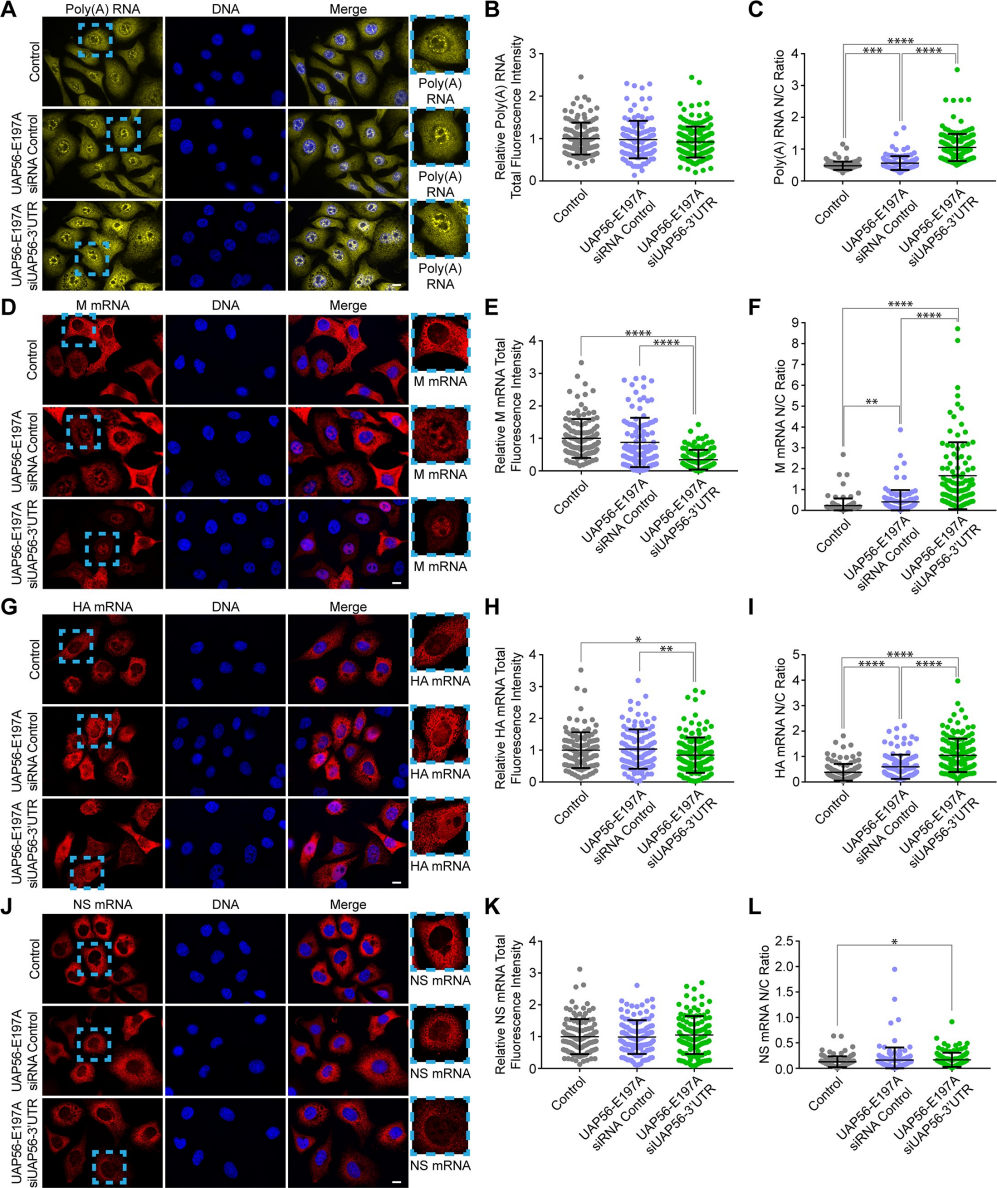
Compound 2 activity phenocopies down-regulation of the mRNA export factor UAP56. A549 cells or A549 cells stably expressing UAP56 E179A mutant were untreated or treated with control siRNA or with siRNA targeting the 3’UTR of UAP56 to knockdown endogenous UAP56 mRNA. Cells were then infected with WSN at MOI 2 for 8h followed by RNA-FISH to detect poly(A) RNA (**A-C**) or smRNA-FISH to detect M (**D-F**), HA (**G-I**), and NS (**J-L**) mRNAs. For **B-C** (C, *n =* 128 cells; UAP56-E197A+siRNA Control, *n =* 117 cells; UAP56-E197A_siUAP56-3’UTR, *n* = 170 cells). For **E-F** (C, *n =* 121 cells; UAP56-E197A+siRNA Control, *n =* 106 cells; UAP56-E197A_siUAP56-3’UTR, *n* = 108 cells). For **H-I** (C, *n =* 124 cells; UAP56-E197A+siRNA Control, *n =* 118 cells; UAP56-E197A_siUAP56-3’UTR, *n* = 151 cells). For **K-L** (C, *n =* 113 cells; UAP56-E197A+siRNA Control, *n =* 119 cells; UAP56-E197A_siUAP56-3’UTR, *n* = 115 cells). Graphs show data points and mean +/- SD. *p<0.05, **p<0.01, ***p<0.001 ****p<0.0001.

To further corroborate these data, we tested the effect of a catalytically inactive mutant of UAP56 (E197A) [31–33] on nuclear export of viral M, HA, NS1, and poly(A) RNA. We have generated cells stably expressing UAP56 (E197A), as we recently reported [34]. These cells were treated with control siRNA or with siRNA that targets the 3’UTR of UAP56 –this siRNA depletes endogenous UAP56 and not UAP56 mutant [34]. The efficiency of this siRNA is shown in Fig 6G and 6H. Control cells and UAP56 (E197A) mutant cells were then subjected to RNA-FISH to label poly(A) RNA (Fig 7A–7C) or infected with WSN followed by smRNA-FISH to detect M, HA, and NS1 mRNAs (Fig 7D–7L) followed by fluorescence microscopy. In the UAP56 mutant cells treated with siRNA control, the total levels of these mRNAs are not altered while nuclear export of M and HA mRNAs is preferentially blocked, poly(A) RNA export is slightly inhibited, and NS1 mRNA export is not altered (Fig 7). When these mutant UAP56 cells were then treated with siRNA against endogenous UAP56, the total levels of M and HA mRNAs were reduced (Fig 7E and 7H) and the levels of poly(A) RNA and NS1 mRNA were not altered (Fig 7B and 7K). On the other hand, nuclear export of M mRNA was severely blocked, poly(A) RNA and HA mRNA export was also inhibited, and NS1 mRNA was only slightly altered (Fig 7C–7L). Taken together, these results show that compound 2 phenocopies partial down-regulation of UAP56 activity as shown by either depleting UAP56 with low levels of siRNA (Fig 6G, 6H and 6J) or by expressing UAP56 mutant in the presence of endogenous UAP56 (UAP56–E197A + siRNA control) (Fig 7). Since UAP56 is a critical mRNA export factor for viral M mRNA, these results further corroborate the screening strategy to identify inhibitors of the M mRNA nuclear export such as compound 2. Additionally, the differential effect of down-regulating UAP56 activity on nuclear export of certain viral mRNAs further emphasize the concept of preferential usage of specific mRNA export factors or adaptors by a subset of mRNAs.

**Fig 8:**
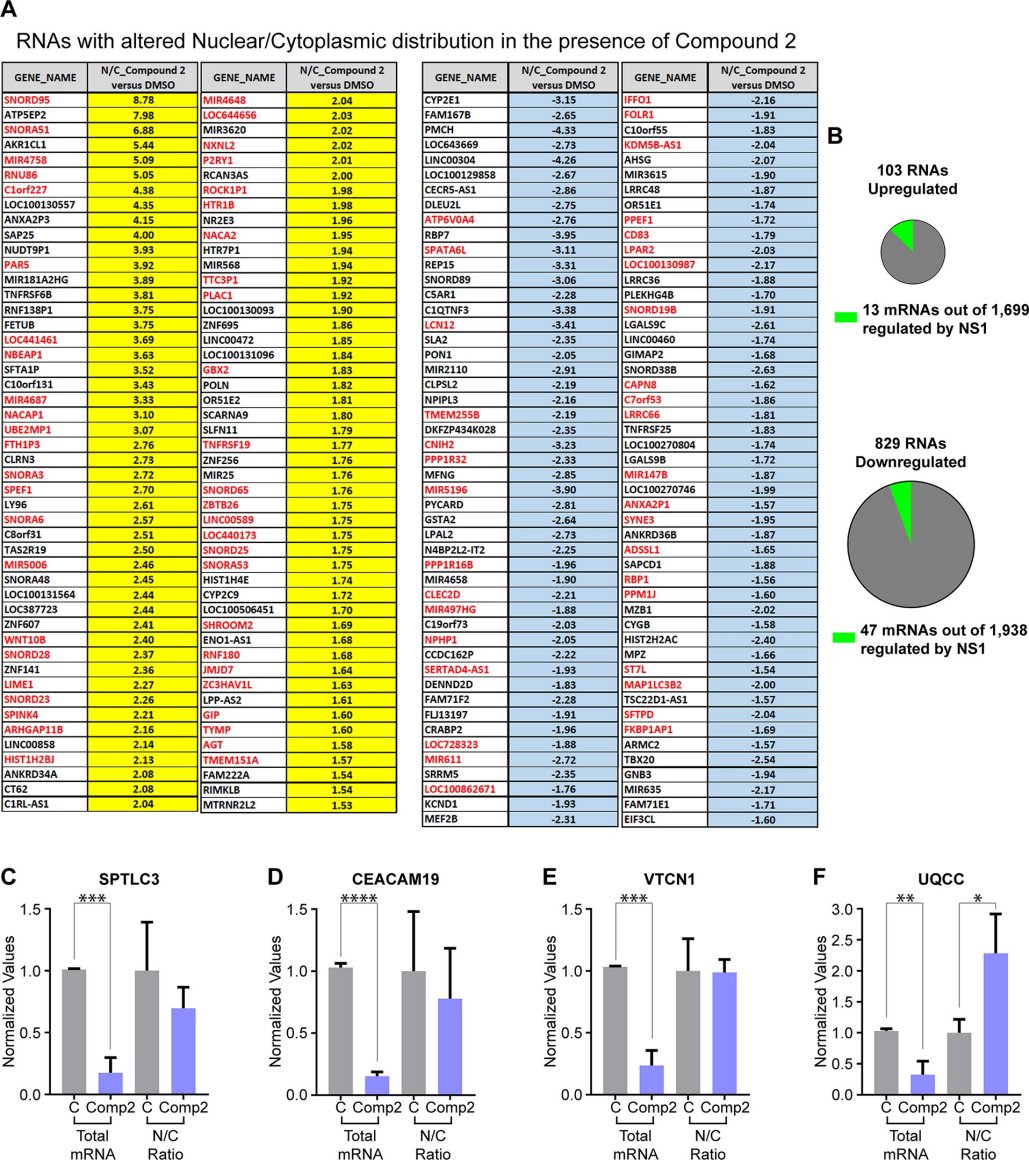
Compound 2 alters the levels and intracellular distribution of a subset of cellular mRNAs. Poly(A) RNA from total cell lysates, nuclear and cytoplasmic fractions untreated or treated with compound 2 was subjected to RNAseq analysis. Two biological duplicates were analyzed and the cut off is 1.5 fold for all analysis. RNAs selected were hits in both samples. (**A**) RNAs that are nuclear retained (yellow) or preferentially exported to the cytoplasm (light blue) are shown. Marked in red are RNAs whose total levels were not altered. Controls for fractionation are shown in S1 Table. (**B**) The number of RNAs that are up-regulated or down-regulated by compound 2 are shown. Marked in green are the number of RNAs known to be regulated by NS1 during infection. The identity of these RNAs are shown in S1 Table. (**C-F**) Selected mRNAs were also analyzed by qPCR to corroborate the RNAseq analysis. Relative mRNA levels and nuclear to cytoplasmic ratios of SPTLC3 (**D**), CEACAM19 (**E**), VTCN1 (**F**), and UQCC (**G**) were determined by qPCR from RNA obtained from total cell lysates, nuclear and cytoplasmic fractions treated with 0.1% DMSO or 2.5 μM compound 2 for 9 h. Three independent experiments were performed. C, control; Comp 2, Compound 2. Graphs show mean +/- SD. *p<0.05, **p<0.01, ***p<0.001 ****p<0.0001.

To quantitatively assess a potential impact of compound 2 on a subset of cellular RNAs and determine their identity, we performed RNA-sequencing (RNA-seq) analysis of purified poly(A) RNA obtained from whole cells, nuclear fractions, and cytoplasmic fractions either treated with DMSO (control) or with 2.5 μM of compound 2 (S1 Table). As expected, RNAs that are known to be retained in nucleus, such as MALAT1, are primarily nuclear, and mRNAs that are distributed in the nucleus and cytoplasm, such as GAPDH mRNA, are shown in both compartments (S1 Table). A total of 19,799 unique RNAs were sequenced and the cutoff was 1.5-fold change to be considered differentially expressed in the presence of compound 2. We show that compound 2 altered the nuclear to cytoplasmic distribution of a small subset of cellular RNAs, including mRNAs and non-coding RNAs (Fig 8A). Among the non-coding RNAs were small nucleolar RNAs (snoRNAs), miRNAs, and long non-coding RNAs. While snoRNAs are not polyadenylated, pre-snoRNA polyadenylation has been shown to link different steps of snoRNA processing [35]. Similarly, pre-miRNAs are polyadenylated and some long non-coding RNAs also have poly(A) tails, explaining their presence in our poly(A) RNA selection. We found that the nuclear to cytoplasmic distribution of 194 RNAs were altered upon compound 2 treatment (Fig 8A). Among these RNAs, 96 were preferentially retained in the nucleus (high nuclear/cytoplasmic ratio) (Fig 8A, yellow) and 98 were more cytoplasmic compared to control cells (Fig 8A, blue). Within the RNAs blocked in the nucleus, 48 out of the 96 RNAs were not altered at their total levels (Fig 8A, gene name marked in red) indicating nuclear export block similar to the viral M and HA mRNAs upon compound 2 treatment (Fig 5G–5L). In the category of preferentially exported to the cytoplasm, 36 out of the 98 cellular RNAs were not affected at their total levels, suggesting enhanced nuclear export (Fig 8A, gene name marked in red). Regarding the additional RNAs that had both altered total levels and nuclear to cytoplasmic ratios, the regulation may or may not involve nuclear transport as other RNA processing steps could be also compromised, which is a topic for future investigation. This RNAseq analysis also revealed the subset of RNAs up-regulated (103 RNAs) and down-regulated by compound 2 (829 RNAs) (Fig 8B). Among these groups, a small number of mRNAs (13 up-regulated and 47 down-regulated) are also known to be regulated by the viral NS1 protein, as shown in infections performed with WSN compared to WSNΔNS1 [36]. In the category of down-regulated RNAs, gene set enrichment analysis (GSEA) showed tyrosine metabolism altered by compound 2 (p-value = 2.23x10^-5^ and a FDR q-value = 4.98x10^-2^). Fig 8C–8F show examples of selected mRNAs whose total levels as well as nuclear and cytoplasmic distribution were assessed by qPCR and were consistent with our RNAseq results. Thus, these results indicate an effect of compound 2 on a subset of RNAs and not on bulk poly(A) RNA.

### Compound 2 inhibits replication of diverse influenza A viruses

**Fig 9:**
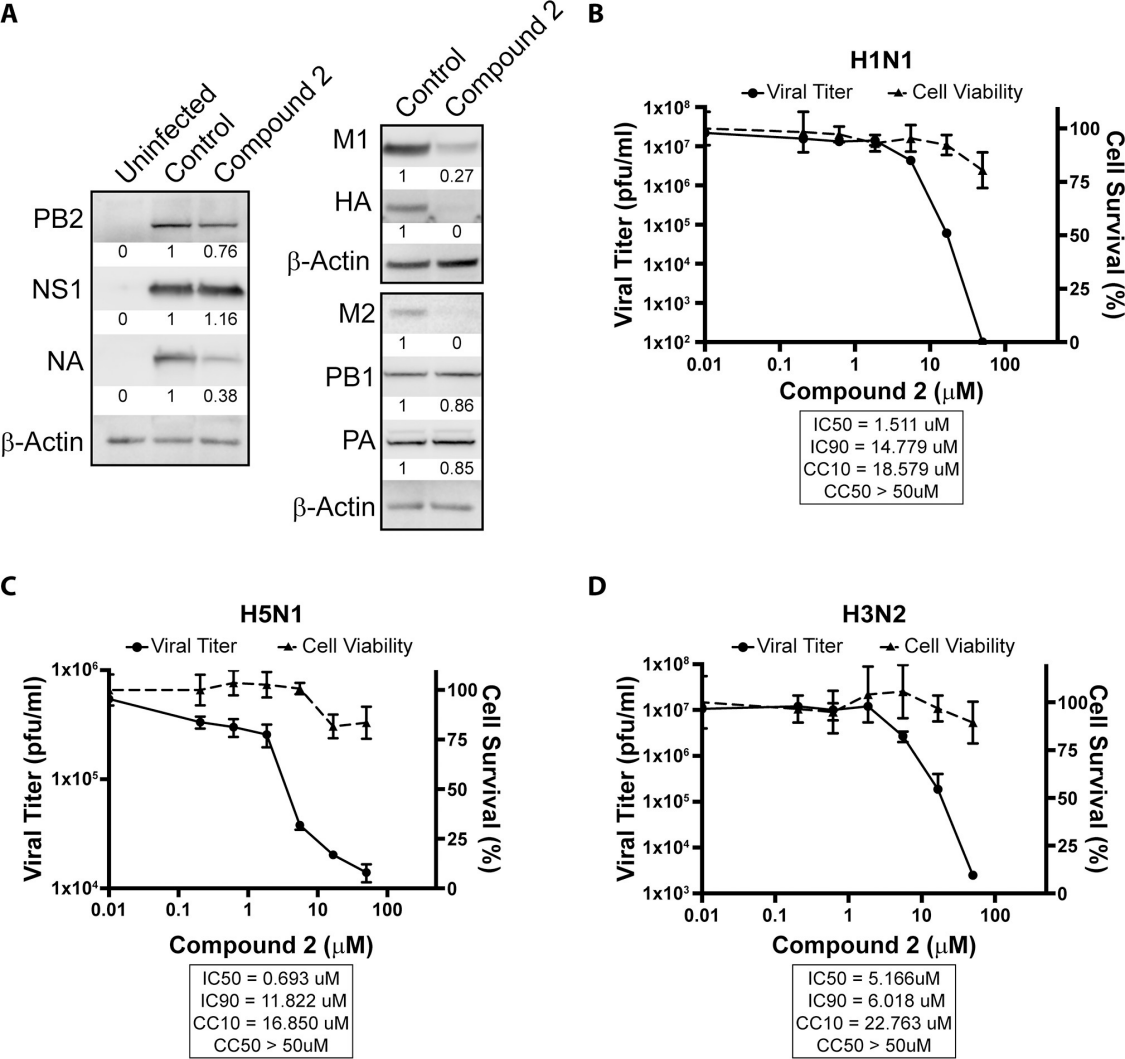
Compound 2 inhibits viral protein production and replication. (**A**) A549 cells were pre-treated with either 0.1% DMSO or 2.5μM compound 2 before infection with A/WSN33 at MOI 2 for 8 h. Cell lysates were subjected to western blot analysis to detect viral proteins including PB1, PB2, PA, NA, NS1, M1, M2, and HA. β-Actin was used as a loading control. This blot is a representative of three independent experiments. (**B-D**) Effect of compound 2 on cell viability and viral replication of (**B**) A/WSN/33 (H1N1), (**C**) A/Vietnam/1203/04 (H5N1), and (**D**) A/Panama/99 (H3N2) influenza A virus strains. Cell viability was determined by the MTT assay in cells treated for 24 h (H1N1 and H5N1) or 48 h (H3N2). Viral titer was determined by plaque assay in cells infected for 24 h (H1N1 and H5N1) or 48 h (H3N2) at MOI 0.01. Three independent experiments were performed. Error bars are SD.

Since nuclear export of key viral mRNAs is blocked by compound 2 and given that these mRNAs encode critical proteins for the virus life cycle, it is expected that viral protein levels and replication would be altered by this compound. Indeed, there is a decrease in the levels of the viral M1 and M2 proteins as well as NA and HA proteins upon 2.5 μM compound treatment (Fig 9A). We then tested compound 2 for inhibition of virus replication and cytotoxicity. As expected, compound 2 inhibited replication of diverse influenza A virus strains at concentrations in which it did not significantly alter cell viability (Fig 9B–9D). Compound 2 also inhibited viral replication in primary human bronchial epithelial cells (S4 Fig). Another compound from our chemical library, ivermectin, is shown as positive control for cytotoxicity at the concentrations used for compound 2 (S5 Fig). In summary, compound 2 preferentially inhibited nuclear export of a subset of mRNAs and further revealed specific requirements for nuclear export of a subset of viral and cellular mRNAs.

## Discussion

Based on our knowledge of viral M mRNA trafficking through host nuclear speckles for splicing and nuclear export [9, 11–13], we designed a high-throughput screening strategy that led to the identification of small molecules that interfered with specific steps of this pathway. Our image-based chemical screen, which uses single-molecule RNA-FISH, identified three classes of inhibitors that either decreased viral M mRNA levels (class 1), or blocked it in the nucleus (class 2), or both (class 3). Our primary HTS assay proved to be quite robust, as exemplified by an average Z’ value of 0.63 for the N/C ratio when comparing the DMSO (vehicle) control to a positive control, DRB. To ensure that we sampled all of the chemical space identified by the screen, we clustered the initial set of hits into chemical series for compounds that decreased the M mRNA fluorescence intensity (552 clusters, intensity reduced > 25%) and for compounds that decreased the N/C ratio (~1300 clusters, N/C ratio > 25%). We then selected cluster representatives from both groups as described above (See Results.) Hit confirmation studies identified ~600 compounds that fell into the three phenotypic classes described above. These compounds were subsequently reviewed for chemical attractiveness (e.g. absence of problematic substructures or PAINS, synthetic tractability, etc.). In this report, we highlight an inhibitor that preferentially prevented nuclear export of a subset of viral mRNAs (class 2), resulting in their accumulation in the nucleoplasm. Since this small molecule (and others like it identified by the screen) did not substantially alter bulk cellular mRNA levels or their intracellular distribution and were not cytotoxic at active concentrations, they may serve as leads for potential antiviral therapy. Therefore, these data revealed a window of opportunity to target a pathway that processes a subset of viral and cellular mRNAs [9, 13]. In addition, compound 2's differential nuclear export inhibition of viral mRNAs and cellular mRNAs demonstrates specific requirements within the mRNA export machinery for nuclear export and provides a tool to distinguish these pathways in future studies.

The differential effect of compound 2 on viral M mRNA nuclear export, phenocopying down-regulation of UAP56 activity, further corroborates its action on the UAP56-NXF1-mediated mRNA export pathway. This would be predicted based on the screening strategy presented here. UAP56 is known to recruit the mRNA export factor Aly/REF to the mRNA, which then binds the mRNA export receptor NXF1•NXT1. This interaction displaces UAP56 from the mRNA and NXF1•NXT1 then docks the mRNP to the nuclear pore complex for export into the cytoplasm [37]. Prior to docking at the nuclear pore complex, the M mRNA is spliced at nuclear speckles and then exported to the nucleoplasm for translocation through the nuclear pore complex [9]. UAP56 is localized at nuclear speckles and in the nucleoplasm [38] and is required for exit of M mRNA from nuclear speckles to the nucleoplasm, as we have previously shown [9]. The localization and export function of UAP56 in the nucleoplasm and at nuclear speckles may involve different factors/adaptors. In contrast to M mRNA [9] and a subset of cellular mRNAs [20–24] whose splicing and/or export occur at nuclear speckles, most cellular mRNAs are spliced in the nucleoplasm prior to being exported from the nucleus. Compound 2 targets the viral M mRNA nuclear export without affecting its splicing at nuclear speckles. Therefore, it is likely that this small molecule is targeting a step between nuclear speckles and the nuclear pore complex, resulting in the accumulation of viral M mRNA throughout the nucleoplasm. Since bulk cellular mRNAs were not substantially affected by the compound at a concentration that it robustly inhibited M and HA mRNA nuclear export, it is possible that this compound is specifically targeting a step or location that affects a subset of cellular mRNAs. In fact, RNAseq analysis shows effect of compound 2 on nuclear export and total levels of a subset of cellular RNAs. This is consistent with the data in which partial depletion of UAP56 or expression of a UAP56 mutant in the catalytic domain in the presence of endogenous UAP56 preferentially blocked viral M and HA mRNA nuclear export without substantially altering NS1 mRNA or bulk cellular mRNAs. These differential effects by partially decreasing the levels of an mRNA export factor reveal a window of opportunity to therapeutically target the mRNA export machinery without inducing major cytotoxicity to the host cell.

Among the subset of cellular mRNAs whose total levels are up-regulated or down-regulated by compound 2 without changes in intracellular distribution, are a few mRNAs known to be regulated by the viral NS1 protein. In the category of up-regulated mRNAs are members of the type-I interferon response system, including IFIT1 and IRF7 [39]. IFN response is known to be suppressed by the NS1 protein therefore both IFIT1 and IRF7 mRNAs are up-regulated in cells infected with the influenza virus lacking NS1 protein [36]. Regarding the down-regulated mRNAs, which were enriched in mRNAs that encode proteins involved in tyrosine metabolism, it is possible that the decrease in tyrosine metabolism inhibits virus replication. Tyrosine is a critical amino acid for viral proteins, such as tyrosine 132 phosphorylation of M1 protein which controls its nuclear import and virus replication [40]. Additionally, virus replication is blocked by receptor tyrosine kinase inhibitors [41]. Furthermore, 47 mRNAs in this down-regulated category are also regulated by NS1. Together, these data suggest that inhibition of influenza virus replication by compound 2 may be a combinatory effect of inhibition of viral mRNA export and induction of antiviral response which, at least in part, involves the type-I interferon system.

Compound 2 is an alkylated mercaptobenzimidazole featuring an aminopyridine amide. No biological activities have been attributed to this compound previously. However, a structurally related series of N-aryl mercaptobenzimidazoles have been described as inhibitors of influenza viruses and myxoviruses [42, 43]. We show that the most potent compound of this series had no effect on M mRNA nuclear export (S6 Fig), indicating that this series operates through a distinct mechanism(s). Accordingly, compound 2 represents an attractive starting point for additional drug discovery efforts. In addition, the screen presented here yielded compounds with various phenotypes–inhibitors of viral M mRNA biogenesis, processing, and/or nuclear export–thus, this strategy expands the landscape for targeting influenza virus at multiple steps of the virus M mRNA intranuclear pathway. As robust viral therapy will likely rely on combination of drugs, this strategy provides multiple leads for drug development. This combinatorial process also contributes to enhance efficacy against diverse viral strains as these compounds may differentially target influenza virus strains. These small molecules are also valuable tools for further understanding new cell biology. They will likely uncover critical regulatory steps and novel factors involved in a yet understudied viral mRNA processing and export pathway.

## Materials and methods

### Ethics statement

The study using embryonated chicken was carried out in strict accordance with recommendations in the Guide for the Care and Use of Laboratory Animals of the National Institutes of Health. The use of embryonated chicken eggs before hatching is not considered animal use. Embryonated eggs were purchased from Charles River Laboratories, inoculated with influenza viruses at day 10, incubated at 37°C for 2 days, and then incubated at 4°C overnight before allantoid fluid harvesting.

### Cell culture

Human lung adenocarcinoma epithelial cells (A549) and MDCK cells, obtained from ATCC (American Type Culture Collection), were maintained in high-glucose DMEM (Gibco), 10% FBS (Sigma), and 100 units/mL Pen/Strep antibiotics at 37°C with 5% CO_2_. Primary human bronchial epithelial cells were cultured as reported [44]. A549 cells stably expressing UAP56 E179A mutant were generated as we recently reported [34].

### Transfections and siRNAs

siRNAs were reverse transfected with A549 cells using RNAiMAX (Invitrogen) in OptiMEM (ThermoFisher) by the manufacturer’s instructions. After 24h transfection, media was replaced with growth media. Knockdown was allowed to continue for 48h before compound treatment or infections occurred. siRNAs used include UAP56 and MISSION siRNA Universal Negative Control #2 (Sigma-Aldrich), ON-TARGETplus siRNAs against SON and ON-TARGETplus Non-targeting Control #2 (Dharmacon, ThermoFisher), and 3’UTR siUAP56 sequence 5’-GCUUCCAUCUUUUGCAUCAUU-3’ (Dharmacon).

### NS1-BP knockout cell line

The NS1-BP gene was knocked out in A549 cells by genome editing using CRISPR–Cas9. In brief, the genomic target oligos (Forward: CACCGTGCTTATGGCCATTCTCACG, Reverse: AAACCGTGAGAATGGCCATAAGCAC) were cloned into a lentiCRISPRv2 vector. The plasmid was co-transfected into HEK293T cells, obtained from ATCC (American Type Culture Collection), with the packaging plasmids pVSVg and psPAX2, generating lentivirus to infect A549 cells. Then, cells were clonally selected using puromycin (1.0 μg/mL) for 7 days followed by 3 days without selection for expansion. Clones were isolated and expanded to generate lysates for western blot analysis using anti-NS1-BP antibody. Candidate clones were subjected to genomic sequencing using amplicons flanking the sgRNA-target site. Growth rates were determined by measuring ATP levels. Cells were tested at 24 h, 48 h, 72 h, and 96 h after plating equal number of NS1-BP^+/+^ and NS1-BP^-/-^ cells. ATP was measured by luminescence using CellTiter-Glo (Promega) according to the manufacturer’s instructions.

### Viruses

Influenza A viruses (A/WSN/33, A/Vietnam/1203/04, A/Panama/99) were generated in embryonated eggs or in MDCK cells after growth from a clonal population of virus at low multiplicity of infection to avoid accumulation of defective virus particles. In MDCK cells, virus was amplified at MOI 0.1–0.001 in infection media containing EMEM (ATCC, 30–2003), 10mM HEPES (Gibco), 0.125% BSA (Gibco), 0.5μg/mL TPCK trypsin (Worthington Biomedical Corporation). Cells were incubated with virus for 1 hour at 37°C, then washed before amplification in infection media. After cell death was observed at 48–72 hours post-infection, supernatants were centrifuged at 1,000 x *g* for 10 minutes to remove cell debris, aliquoted, and stored at -80°C. All virus stocks are controlled for an appropriate ratio of HA/PFU titer and sequenced by RNAseq to confirm the full sequence of the virus.

### Viral replication and cytotoxicity assays

A549 cells were infected with A/WSN/33 and A/Vietnam/1203/04 at MOI 0.01, or with A/Panama/99 at MOI 0.1 in the absence or presence of compound 2 at concentrations depicted in the figures. Supernatants were collected from each condition 24 h post-infection and viral particles were tittered by plaque assay as following: MDCK cells were seeded in 6-well plates to reach confluency the next day. At confluency, ten-fold serial dilutions of each sample’s supernatant were diluted in PBS containing 100 units/mL Pen/Strep antibiotics, 0.2% BSA, 0.9 mM CaCl_2_, and 1.05 mM MgCl_2_. After infection with each dilution, cells were overlaid with a 1:1 mixture of 2X L-15 media and 2% Oxiod Agar (Final concentration of 1X L-15 media and 1% Agar). Plaques formed at 24 h for A/WSN/33 and A/Vietnam/1203/04, or 48 h for A/Panama/99 were counted and titers determined. Primary human bronchial epithelial cells were infected with A/WSN/33 at MOI 0.1 for 24 h in the absence or presence of compound 2 at depicted concentrations. Supernatants were subjected to plaque assays as described above.

Cytotoxicity was also performed using the MTT assay (Roche), according to the manufacturer’s instructions, concurrent with viral replication assay.

### smRNA-FISH

smRNA-FISH was performed as previously described [9], which includes the sequences of M1 and NS1 probes except for the HA probes that are listed below. Briefly, cells were grown on glass coverslips (Fisherbrand, Fisher Scientific) coated with 1mL of 0.1% gelatin (Sigma-Aldrich). Cells were fixed with 4% paraformaldehyde (PFA, Electron Microscopy Sciences) in PBS for 15 min before incubation in 70% ethanol for 12 h at 4°C. Coverslips were then placed in wash buffer for 5 min, containing Nuclease Free Water, 2x SSC Buffer (Sigma), and 10% formamide (Sigma). The coverslips where then removed and incubated in hybridization buffer containing FISH probe. Hybridization occurred at 37°C for 4 h, then cells were washed with wash buffer for 30 min at 37°C. Coverslips were then washed twice for 5 min in PBS and stained with 1 μg/ml Hoechst 33258 (Molecular Probes/Life Technologies) for 10 min. Coverslips were briefly washed with PBS before mounting in ProLong Gold antifade reagent (Life Technologies).

#### HA mRNA Probes

Forty-eight, 20nt DNA probes labeled with Quasar 570 (BIOSEARCH TECHNOLOGIES) were designed to hybridize with the Influenza WSN full length HA mRNA (Table 1).

**Table 1:** HA mRNA Probes.

Probe #	Probe (5'-> 3')
1	catattgtgtctgcatctgt
2	ttgagttgttcgcatggtag
3	gccacattcttctcgaatat
4	gtcttcgagcaggttaacag
5	ttacatagtttcccgttgtg
6	caattgtagtggggctattc
7	catccggtgatgttacattt
8	tgagtcgcattctggatttc
9	cattctcagagtttggtgtt
10	tcagttcctcatagtcgatg
11	gatactgagctcaattgctc
12	ccatgaactttccttgggaa
13	gagcatgatactgttactcc
14	gtaaaaactgctttttcccc
15	ttcgtcagccatagcaaatt
16	aattggtcagctttgggtat
17	tttccctttattgttcacat
18	tgatgaacaccccatagtac
19	gggtgaatctcctgttataa
20	cccatgttgatcttttactt
21	gcaaggtccagtaatagttc
22	tattagattaccagttgcct
23	tcagtgcgaaagcataccat
24	tgatgatgccggactcaaac
25	tcatgcattgacgcgtttga
26	gtgtttgacacttcgtgtta
27	gattgctgtttatagatccc
28	gactgggtgtatattctgga
29	tgacatattttgggcactct
30	gtaaccatcctcaatttggt
31	ggatgggatgtttcttagtc
32	ctccaaatagacctctgtat
33	cccctcaataaaaccagcaa
34	aaccataccatccatctatc
35	ttttttgatccgctgcatag
36	tttgtaatcccgttaatggc
37	ctcgataacagagttcacct
38	tgtccaaatgtccagaaacc
39	ggctttttactttctcgtac
40	tccgatttctttggcattat
41	tcattgtcacacttgtggta
42	aagtcccatttcttacactt
43	ctatcttttccctgttcaac
44	cccattgattccaatttcac
45	tggcgacagttgagtagatc
46	gagaccaaaagcaccagtga
47	acatccagaaactgattgcc
48	atgcatattctgcactgcaa

### High-throughput screen and statistics

To identify chemical inhibitors of viral M mRNA processing and nuclear export, A549 cells were treated with 232,500 chemical compounds available from the University of Texas Southwestern Medical Center High Throughput Screening core facility. Cells were treated with 2.5 μM compound for 30 minutes and incubated at 37°C in 5% CO_2_. Cells were then infected with influenza A/WSN/33 virus at MOI of 2 and returned to incubation as before. At 7.5 hours post-infection, cells were fixed with 4% paraformaldehyde and subjected to RNA-FISH. To localize M mRNA, we used forty-five FISH probes labeled with Quasar 570 that cover the entire M mRNA segment, as previously reported [9]. Nuclei were stained with 1 μg/mL Hoechst 33342 dye. M mRNA distribution between the nucleus and the cytoplasm was detected using the IN Cell Analyzer 6000 (GE Healthcare, Marlborough MA). Multiple fields per well were taken at 20X magnification using the Hoechst and dsRed widefield fluorescence filters. Image analysis was performed in a GE IN Cell Analyzer Workstation 3.7.3 (GE Healthcare) using the multi-target analysis template. Individual nuclei were segmented using a top-hat filter on the Hoechst channel with the default sensitivity setting. For samples detecting the M1 mRNA, the cell body was segmented using the region growing method on the M1 mRNA channel. This method uses the nuclei as the seed and then expands outwards until the edge of the stain is reached. For samples detecting poly(A) RNA, the poly(A) RNA channel was instead used to define the cell body region using the region growing method. For each segmented nucleus and cell pair, the mean and total signal intensities of the nuclear and cytoplasmic chambers were calculated for the poly(A) RNA (where applicable) and M1 mRNA channels. The mean nuclear to mean cytoplasmic (N/C) ratio was then calculated for both mRNA probes for each cell. Finally, the average N/C ratios per well were calculated and used for hit identification. The results were imported into the GeneData Screener^™^ (Basel, Switzerland; version 13.0.6) software analysis suite to normalize and summarize the overall M mRNA intensity as well as nuclear to cytosolic ratio in terms of a Z-score as previously described [45, 46].

In the primary screen, compounds with a robust Z-score of less than -3 for intensity were considered hits affecting virus replication. Compounds with a Z-score greater than 3 in the nuclear/cytosolic ratio were selected as hits for inhibition of nuclear export. Any compound that lowered the nuclear count to a Z-score of -3 or lower was considered cytotoxic and not included in follow-up experiments. Compounds (1,125) that had the highest activity were selected for confirmation and retested in triplicate at a compound concentration of 2.5 μM. All imaging confirmation and follow-up assays included a bulk poly(A) RNA probe linked to Quasar 670 for FISH imaging. As with the M mRNA probe, total intensity and N/C ratio were also measured for the poly(A) RNA probe. The 600 compounds with the highest activity from the confirmation assay were subjected to 12 point dose response curves ranging from 0.5 nM to 50 μM at 0.5 log dose intervals. Of the 600 compounds tested, 413 compounds had a measureable effect on bulk poly(A) RNA and were excluded from further testing. The remaining 187 compounds that inhibited viral mRNA nuclear export and/or decreased viral mRNA levels but had no substantial effect on the host cell poly(A) RNA were categorized into 3 major phenotypes. These include 22 compounds that retained viral M mRNA in the nucleus, 33 compounds that decreased viral M mRNA levels, and 132 compounds that decreased overall levels and inhibited nuclear export of viral M mRNA. Clustering analysis of confirmed hits was performed with Pipeline Pilot v16 (Biovia, Inc.) using ECFP4 fingerprints [47].

### Image quantification and statistics

Total cell fluorescence intensity or fluorescence intensity in the nucleus and cytoplasm analysis for Figs 1, 5 and 6 and S6 Fig. Images deconvolved with AutoQuant software were analyzed using Imaris (Bitplane). The Surfaces tool was used to segment fluorescence within the cytoplasm and nucleus of each cell quantified. Statistical analyses for imaging studies and qPCR data in the figures mentioned above were performed using the two-sample, two-tailed, *t*-test.

### Compounds

Compound 2-thiobenzimidazole was initially purchased from TimTec (HTS04595) as well as synthesized in-house. JMN3-003 was synthesized as previously described [42]. All compounds were dissolved in dimethylsulfoxide (DMSO). Compound 2 was synthesized and characterized as following (See S7 Fig): a mixture of 2-mercaptobenzimidazole (30.0 mg, 0.2 mmol, 1.0 equiv.) and crushed potassium hydoxide (11.2 mg, 0.2 mmol, 1.0 equiv.) in 2 mL of ethanol was kept at reflux for 2 hours. The reaction mixture was cooled down to room temperature, N-(5-bromopyridin-2-yl)-2-chloroacetamide (49.9 mg, 0.2 mmol, 1.0 equiv.) was added, and the reaction was stirred for overnight. The resulting reaction mixture was concentrated under reduced pressure. 2.0 mL of saturated ammonium chloride solution and 2.0 mL of dichloromethane were added to the residue. The organic layer was separated, washed with 2.0 mL of brine, then dried over sodium sulfate, filtered and concentrated under reduced pressure. The crude was further purified by silica gel chromatography using 60% of ethyl acetate in hexane to afford 54 mg white solid as product, yield 74%.

### ^1^H NMR (CDCl_3_, 400 MHz)

δppm 8.31–8.24 (m, 1H), 8.03 (d, J = 8.8 Hz, 1H), 7.72 (ddd, J = 8.9, 2.8, 1.5 Hz, 1H), 7.48 (br, 2H), 7.20–7.08 (m, 3H), 4.03 (s, 2H).

### ^13^C NMR (CDCl_3_, 400 MHz)

δppm 168.23, 149.92, 149.48, 148.78, 140.57, 122.95, 122.35, 115.53, 114.86, 109.97, 36.24.

### MS

MS (ESI) *m/z* = 363.0 ([M+H]^+^), C_14_H_11_BrN_4_OS requires 363.0

### Measurement of cellular ATP levels

ATP was measured by luminescence using the CellTiter-Glo kit (Promega) according to the manufacturer’s instructions.

### RNA purification and RT-qPCR

Total RNA was isolated from A549 cells using the RNeasy Plus Mini Kit (Qiagen) and reverse transcribed into cDNA by SuperScript II reverse transcriptase (Invitrogen), each according to the manufacturers’ protocols. Samples were then amplified in a LightCycler 480 quantitative real-time PCR (qPCR) system (Roche) using SYBR Green I (Roche) and sequence specific primers.

RT-PCR Primer Sequences:

M1 Forward: ATCAGACATGAGAACAGAATGG

Reverse: TGCCTGGCCTGACTAGCAATATC

M2 Forward: CGAGGTCGAAACGCCTATCAGAAAC

Reverse: CCAATGATATTTGCTGCAATGACGAG

NS1 Forward: TGGAAAGCAAATAGTGGAGCG

Reverse: GTAGCGCGATGCAGGTACAGAG

NS2 Forward: CAAGCTTTCAGGACATACTGATGAG

Reverse: CTTCTCCAAGCGAATCTCTGTAGA

HA Forward: TCTATTTGGAGCCATTGCTGG

Reverse: TGCTTTTTTGATCCGCTGCA

18S Forward: GTAACCCGTTGAACCCCATT

Reverse: CCATCCAATCGGTAGTAGCG

β-actin Forward: CCGCGAGAAGATGACCCAGAT

Reverse: CGTTGGCACAGCCTGGATAGCAACG

SPTLC3 Forward: GGAATTGGAACCCTGTTTGGC

Reverse: GTCTCTGATTCGCATGTAAAGGT

CEACAM19 Forward: GCCCAGCCTACAGACAGTG

Reverse: GCAGCAAGAGATCCAATGATGG

VTCN1 Forward: TCTGGGCATCCCAAGTTGAC

Reverse: TCCGCCTTTTGATCTCCGATT

UQCC Forward: GGAGAAAACTGACTTCGAGGAAT

Reverse: TCCAGACGTGGAGTAGGGTTA

UAP56 Forward: CTTTGAGCATCCGTCAGAAGT

Reverse: AGTGTGACACATCACCAGTACA

### Cell fractionation and RNAseq analysis

Cells were treated with 0.1% DMSO or 2.5 μM compound 2 for 9 hours. Nuclear and cytoplasmic fractions were obtained using the NE-PER Nuclear and Cytoplasmic Extraction Reagents (Thermo Fisher Scientific). Controls are discussed in S1 Table. Total RNA was isolated from total cell lysates, nuclear and cytoplasmic fractions using the RNeasy Plus Mini Kit (Qiagen). RNA samples were then analyzed in the Agilent 2100 Bioanalyzer to determine RNA quality (RIN Score 8 or higher). RNA concentration was determined using the Qubit fluorometer. A TruSeq Stranded Total RNA LT Sample Prep Kit (Illumina) was used to prepare 4 μg of DNAse-treated RNA for poly(A) RNA purification and fragmentation before strand specific cDNA synthesis. cDNA libraries were a-tailed and ligated to indexed adapters. Samples were then PCR amplified and purified with Ampure XP beads and validated with the Agilent 2100 Bioanalyzer. Samples were quantified again by Qubit before being normalized and pooled to be run on the Illumina HiSeq 2500 using SBS v3 reagents. Raw FASTQ files were analyzed using FastQC v0.11.2 [48] and FastQ Screen v0.4.4 [49], and reads were quality-trimmed using fastq-mcf (ea-utils/1.1.2–806) [50]. The trimmed reads were mapped to the hg19 assembly of the human genome (the University of California, Santa Cruz, version from igenomes) using STAR v2.5.3a [51]. Duplicated reads were marked using Picard tools (v1.127; **https://broadinstitute.github.io/picard/**), the RNA counts generated from FeatureCounts [52] were TMM normalized [53], and differential expression analysis was performed using edgeR [53]. Expression data is represented as TPM (Transcripts per Million). Genes with mRNA TPM values of zero in either the control or experiment conditions were removed from the analysis. Log2 of the average TPM values for the remaining genes of each condition (total, nuclear, and cytoplasmic) were calculated. Only mRNAs with Log2TPM > -1 were considered for further analysis to remove experimental background noise. The TPM readings of the experiment compared with control samples were used to calculate the positive and negative fold changes from their ratios. The differentially expressed mRNAs with fold changes of + or– 1.5 FC were subjected to GSEA to obtain the enriched pathways.

### Gene Set Enrichment Analysis (GSEA)

Pathway and network analysis were conducted using Gene Set Enrichment Analysis (GSEA) [54] software and the functional datasets were CP: Canonical pathways from the MSigDB [55, 56].

### Western blot

Cell lysis was performed in 250mM Tris HCl pH 6.8, 40% Glycerol, and 8% SDS. Western blot was performed as previously described [12]. Antibodies used in this study to detect viral proteins include Influenza A virions (Meridian Life Science B65141G), M1 and M2 (Thermo MA1-082), NA (GeneTex GTX125974), PA (GeneTex GTX118991), PB1 (Santa Cruz sc-17601), PB2 (Santa Cruz sc-17603), and NS1 (a gift from J.A. Richt, National Animal Disease Center, Iowa) [57]. Antibodies against cellular proteins include β-actin (Sigma A5441) and UAP56 [Anti-BAT1 (C-TERMINAL antibody produced in rabbit, Millipore SAB1307254). Horseradish peroxidase (HRP)-conjugated secondary antibodies include donkey anti-rabbit, sheep anti-mouse (GE Healthcare NA934V and NA931V, respectively), and donkey anti-goat (Jackson Immunoresearch 705–035003). Quantification of protein band intensity was performed using Image Studio software (LI-COR Imaging). Each protein band was normalized to its corresponding loading control. Values listed below each band represent relative band intensity to its corresponding control.

## Supporting information

S1 Fig Growth rate of NS1-BP knockout cells compared to wild-type cells. Cell growth of NS1-BP wild-type and knockout cells was monitored at 24, 48, 72, and 96 hours as determined by CellTiter-Glo. Four independent experiments were performed. Graph shows mean +/- SD. ***p<0.001, ****p<0.0001.
(TIF)

S2 Fig Cluster analysis of confirmed hits. The 187 compounds identified for follow up studies are the most active members of 187 clusters. Within each active cluster, there are related analogs with lesser activity. In this figure, we show the 187 clusters (arbitrarily numbered 1 to 187) on the x-axis and the number of related analogs for each cluster plotted on the y-axis. Cluster size ranged from 1 to 32 members. Singleton clusters comprised 31% of the structural clusters (chemotypes). Compound 2 is a member of cluster 164 (indicated in red), which has 5 members. Clustering was performed with Pipeline Pilot v16 (Biovia, Inc.) using ECFP4 fingerprints.
(TIF)

S3 Fig Control for cell fractionation shown in Fig 6. A549 cells were treated with 1 nM or 20 nM siRNA targeting the 3’UTR of the UAP56 mRNA or with control siRNA and then infected with WSN at MOI 2 for 8h. Purified RNA from total cell extract (**A**) or nuclear and cytoplasmic fractions (**B**) was subjected to qPCR to detect MALAT1 (a long non-coding RNA localized in the nucleus) as a nuclear marker. (C) Purified RNA from **A** was also used to detect total levels of 18S RNA or determine its nuclear to cytoplasmic distribution (D). 18S RNA is preferentially localized in the cytoplasm. Three independent experiments were performed. Graphs show mean +/- SD. Cyto, cytoplasm; Nuc, nucleus.
(TIF)

S4 Fig Compound 2 inhibits influenza virus replication in primary human bronchial epithelial cells at non-toxic concentrations. (**A**) Viral titer was determined by plaque assay in primary human bronchial epithelial cells (HBEC) infected with A/WSN/33 for 24 h in the absence or presence of compound 2 at the depicted concentrations. (**B**) Cell viability was monitored at 24 h after treatment with 0.1% DMSO or compound 2 at the depicted concentrations using CellTiter-Glo. Three independent experiments were performed. Graph shows mean +/- SD. **p<0.01. ***p<0.001, ****p<0.0001
(TIF)

S5 Fig Positive control for compound cytotoxicity. A549 cells were incubated with ivermectin, a compound present in our chemical library, at the depicted concentrations for 48 h. Cell viability was determined by the MTT assay. Three independent experiments were performed. Graph shows mean +/- SD. ***p<0.001.
(TIF)

S6 Fig Compound JMN3-003 (N-aryl mercaptobenzimidazole) does not inhibit viral mRNA nuclear export. (**A**) Structure of compound JMN3-003. (**B**) smRNA-FISH followed by fluorescence microscopy was performed to detect M mRNA in cells treated with 0.1% DMSO or 2.5μM JMN3-003. These treatments started 1 hour before infection with WSN at MOI 2 for 8 h. Total fluorescence intensity (**C**) or nuclear to cytoplasmic fluorescence intensity (N/C ratio) (**D**) of M mRNA was quantified for images in **B**. For both **C** and **D** (C, *n =* 123 cells; JMN3-003, *n =* 141 cells). Graphs show data points and mean +/- SD. ****p<0.0001. This compound decreased total viral M mRNA levels but did not retain viral M mRNA in the nucleus as compound 2.
(TIF)

S7 Fig Compound 2 synthesis. Compound 2 is a 2-((1H-benzo[d]imidazole-2-yl)thio)-N-(5-bromopyridin-2-yl) acetamide. See details in the methods section.
(TIF)

S1 Table Raw data and analysis of RNAseq data presented in Fig 8. Tab 1: Raw Data: RNAseq TPM values of all RNAs mapped in the genome are listed. RNAs obtained from total cell lysates, cytoplasmic and nuclear fractions of cells treated with DMSO (0.1%) or compound 2 (2.5 μM) are shown. Tab 2: Fractionation Controls: TPM values of primarily nuclear mRNAs are shown to validate the nuclear-cytoplasmic fractionation. RNAs such as GAPDH listed in Tab1 show the expected distribution in the nucleus and cytoplasm. This is further corroborated by smRNA-FISH detecting GAPDH mRNA in the nucleus and cytoplasm (Fig 5B and 5E). Tab 3: RNAs up-regulated > 1.5 fold by compound 2 over DMSO control are listed. Tab 4: RNAs up-regulated by compound 2 (from Tab 3) which overlap with RNAs up-regulated in the absence of NS1 during infection. In the latter, cells infected with virus lacking NS1 are compared to cells infected with wild-type virus [36]. Tab 5: RNAs down-regulated < -1.5 fold by compound 2 compared to DMSO control are listed. Tab 6: RNAs down-regulated by compound 2 (from Tab 5) which overlap with RNAs down-regulated in the absence of NS1 during infection. In the latter, cells infected with virus lacking NS1 are compared to cells infected with wild-type virus [36]. Tab 7: mRNAs that are blocked in the nucleus or preferentially exported to the cytoplasm upon compound 2 treatment compared to DMSO control. mRNAs that are nuclear blocked > 1.5 fold change or show enhanced nuclear release < -1.5 fold change are listed.
(XLSX)
