# Supplementary figures and images for: Chemical intervention of influenza virus mRNA nuclear export
Source: PLoS Pathog. 2020 Apr 2;16(4):e1008407. doi: 10.1371/journal.ppat.1008407 (PMC7117665; doi:10.1371/journal.ppat.1008407)

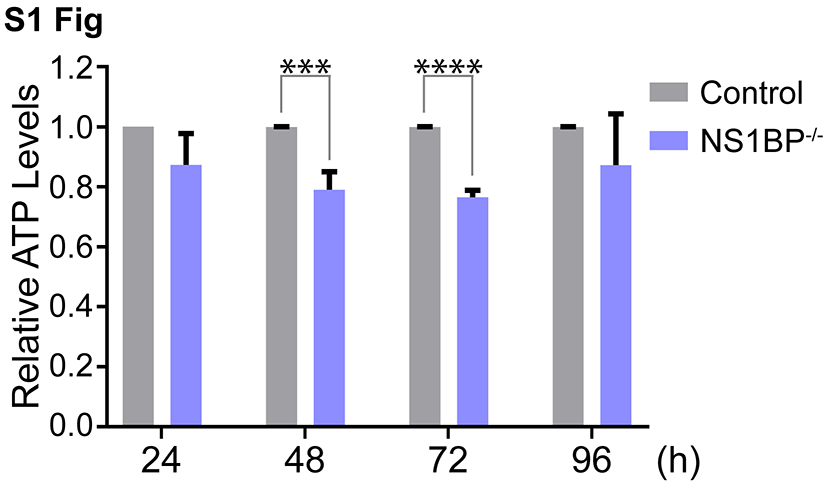

Supplement: S1 Fig — Cell growth of NS1-BP wild-type and knockout cells was monitored at 24, 48, 72, and 96 hours as determined by CellTiter-Glo. Four independent experiments were performed. Graph shows mean +/- SD. ***p<0.001, ****p<0.0001. (TIF) [file ppat.1008407.s001.tif]

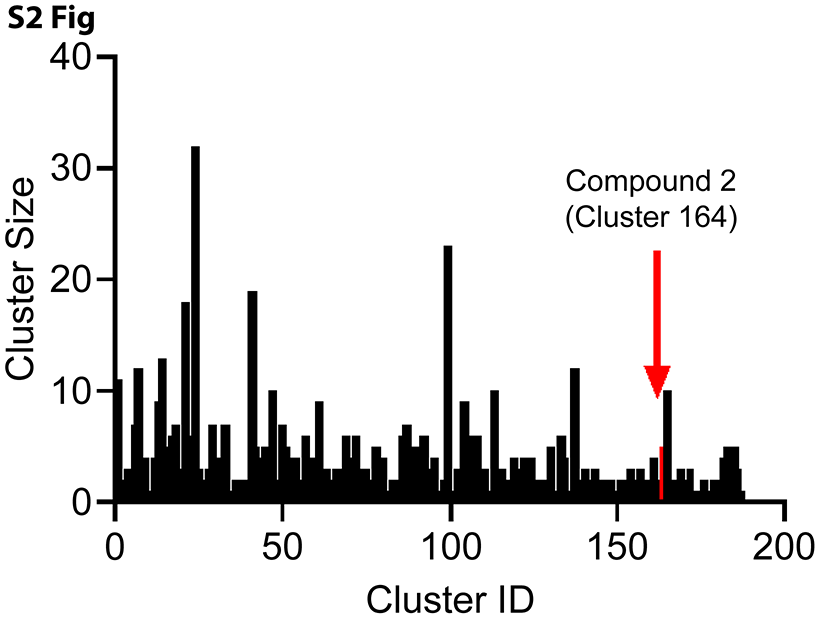

Supplement: S2 Fig — The 187 compounds identified for follow up studies are the most active members of 187 clusters. Within each active cluster, there are related analogs with lesser activity. In this figure, we show the 187 clusters (arbitrarily numbered 1 to 187) on the x-axis and the number of related analogs for each cluster plotted on the y-axis. Cluster size ranged from 1 to 32 members. Singleton clusters comprised 31% of the structural clusters (chemotypes). Compound 2 is a member of cluster 164 (indicated in red), which has 5 members. Clustering was performed with Pipeline Pilot v16 (Biovia, Inc.) using ECFP4 fingerprints. (TIF) [file ppat.1008407.s002.tif]

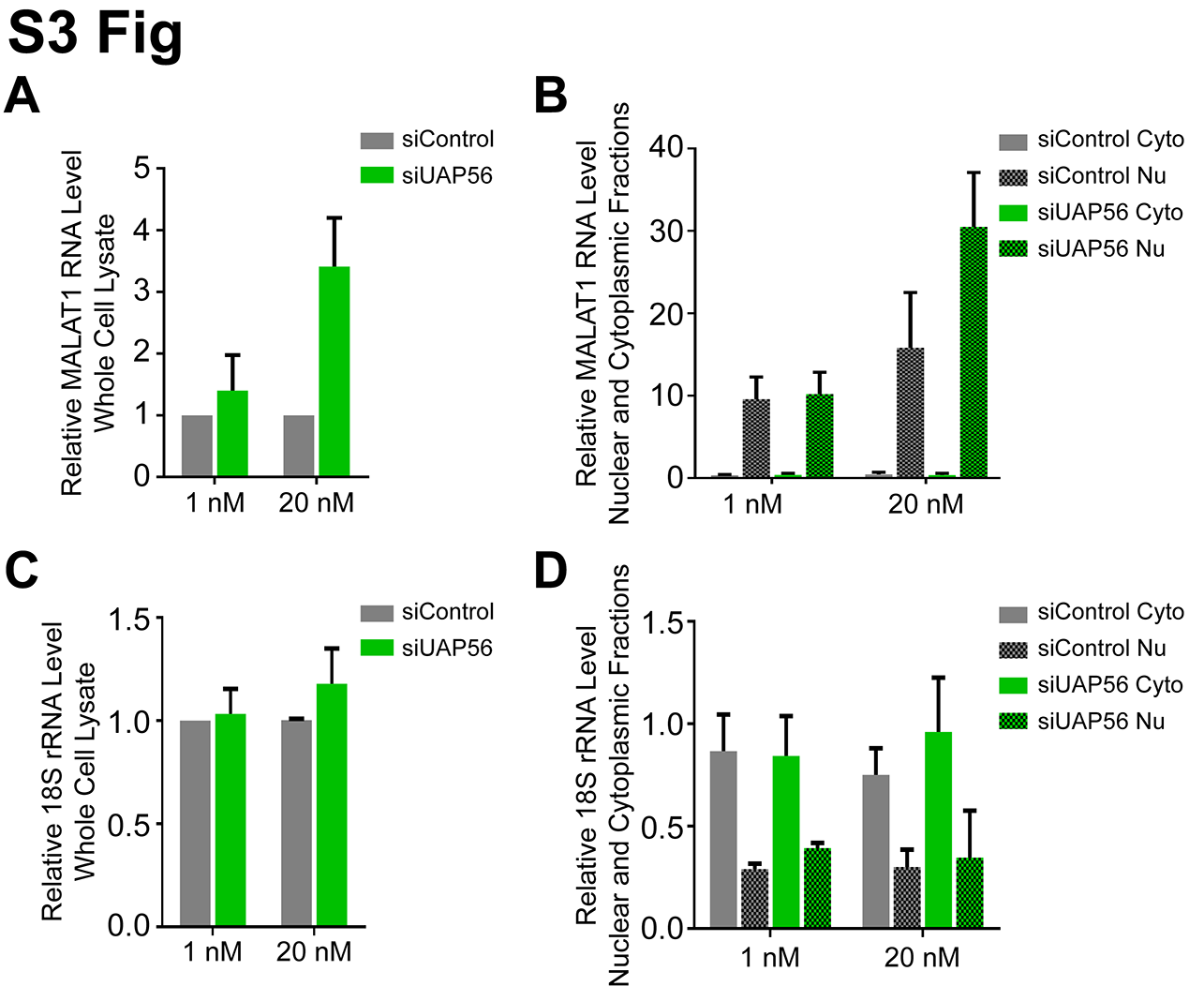

Supplement: S3 Fig — A549 cells were treated with 1 nM or 20 nM siRNA targeting the 3’UTR of the UAP56 mRNA or with control siRNA and then infected with WSN at MOI 2 for 8h. Purified RNA from total cell extract (A) or nuclear and cytoplasmic fractions (B) was subjected to qPCR to detect MALAT1 (a long non-coding RNA localized in the nucleus) as a nuclear marker. (C) Purified RNA from A was also used to detect total levels of 18S RNA or determine its nuclear to cytoplasmic distribution (D). 18S RNA is preferentially localized in the cytoplasm. Three independent experiments were performed. Graphs show mean +/- SD. Cyto, cytoplasm; Nuc, nucleus. (TIF) [file ppat.1008407.s003.tif]

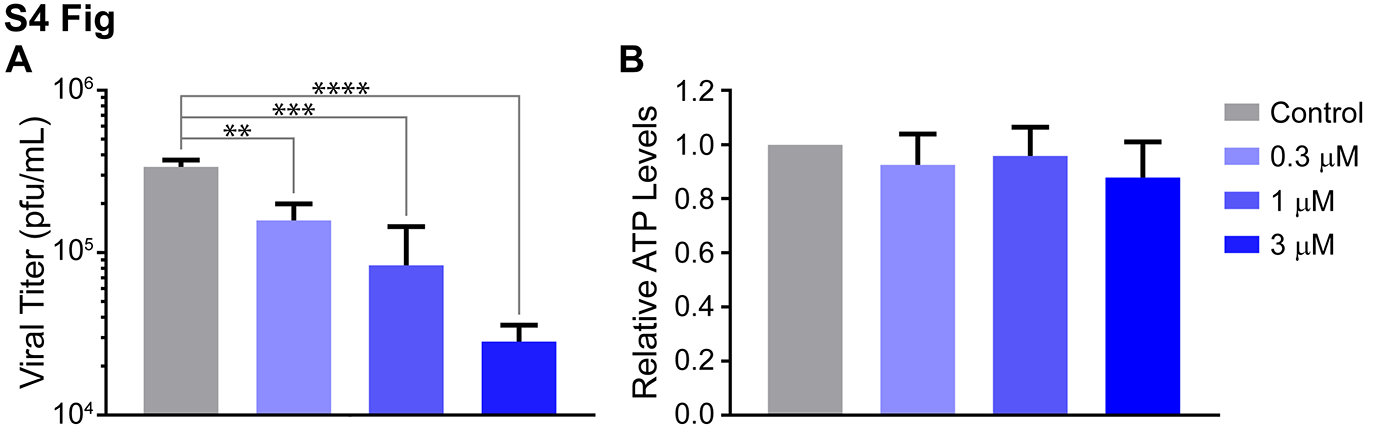

Supplement: S4 Fig — (A) Viral titer was determined by plaque assay in primary human bronchial epithelial cells (HBEC) infected with A/WSN/33 for 24 h in the absence or presence of compound 2 at the depicted concentrations. (B) Cell viability was monitored at 24 h after treatment with 0.1% DMSO or compound 2 at the depicted concentrations using CellTiter-Glo. Three independent experiments were performed. Graph shows mean +/- SD. **p<0.01. ***p<0.001, ****p<0.0001 (TIF) [file ppat.1008407.s004.tif]

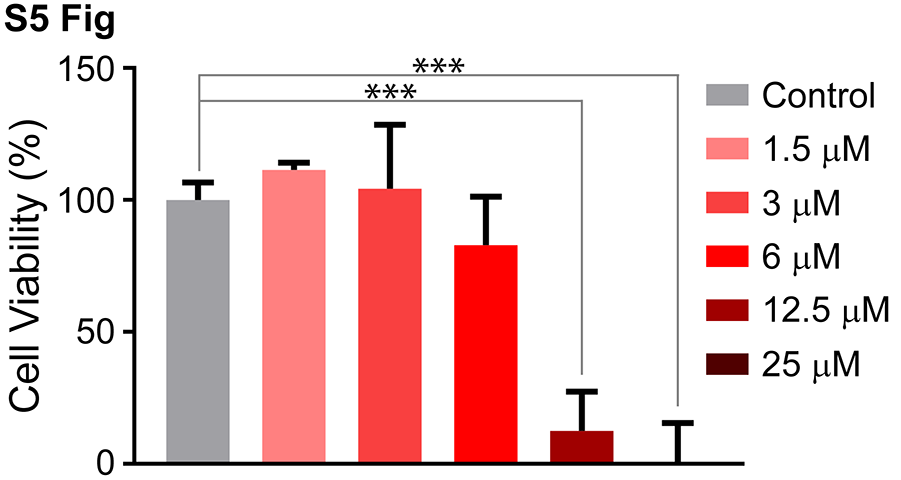

Supplement: S5 Fig — A549 cells were incubated with ivermectin, a compound present in our chemical library, at the depicted concentrations for 48 h. Cell viability was determined by the MTT assay. Three independent experiments were performed. Graph shows mean +/- SD. ***p<0.001. (TIF) [file ppat.1008407.s005.tif]

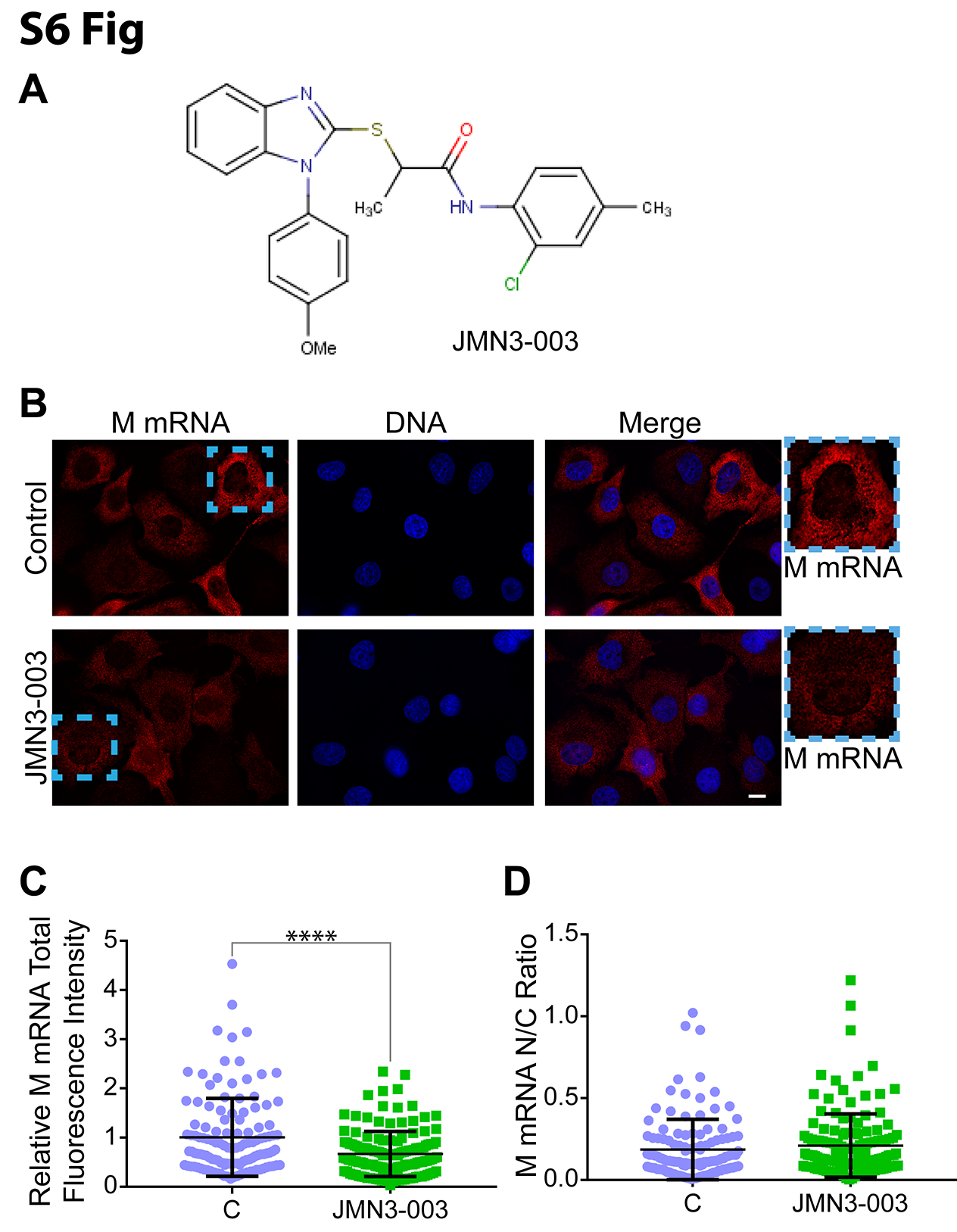

Supplement: S6 Fig — (A) Structure of compound JMN3-003. (B) smRNA-FISH followed by fluorescence microscopy was performed to detect M mRNA in cells treated with 0.1% DMSO or 2.5μM JMN3-003. These treatments started 1 hour before infection with WSN at MOI 2 for 8 h. Total fluorescence intensity (C) or nuclear to cytoplasmic fluorescence intensity (N/C ratio) (D) of M mRNA was quantified for images in B. For both C and D (C, n = 123 cells; JMN3-003, n = 141 cells). Graphs show data points and mean +/- SD. ****p<0.0001. This compound decreased total viral M mRNA levels but did not retain viral M mRNA in the nucleus as compound 2. (TIF) [file ppat.1008407.s006.tif]

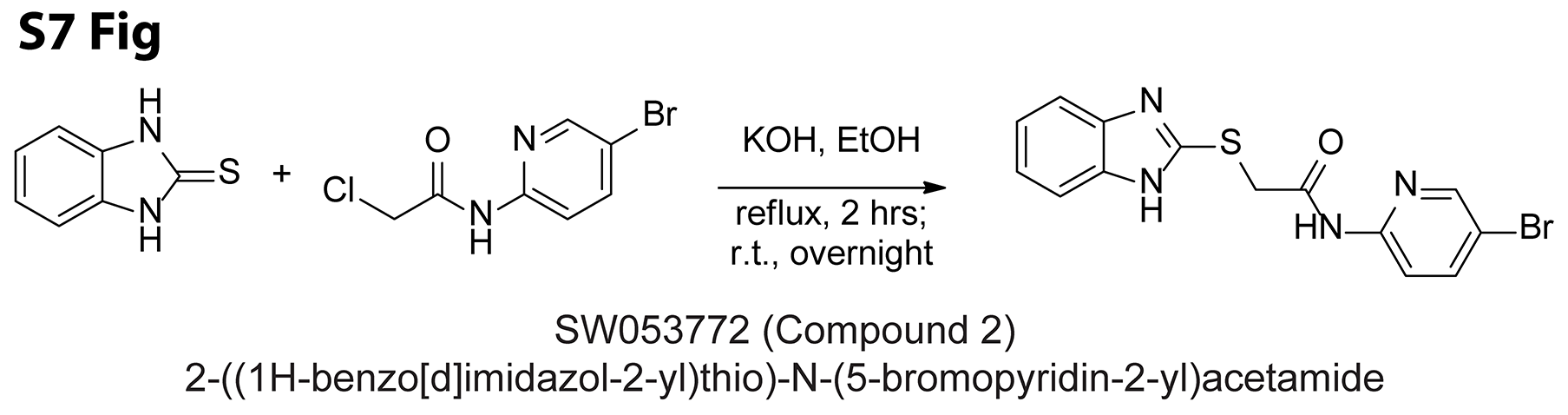

Supplement: S7 Fig — Compound 2 is a 2-((1H-benzo[d]imidazole-2-yl)thio)-N-(5-bromopyridin-2-yl) acetamide. See details in the methods section. (TIF) [file ppat.1008407.s007.tif]
